# Nitrites: An Old Poison or a Current Hazard? Epidemiology of Intoxications Covering the Last 100 Years and Evaluation of Analytical Methods

**DOI:** 10.3390/toxics11100832

**Published:** 2023-10-01

**Authors:** Kaja Tusiewicz, Patryk Kuropka, Elżbieta Workiewicz, Olga Wachełko, Paweł Szpot, Marcin Zawadzki

**Affiliations:** 1 Department of Forensic Medicine, Wroclaw Medical University, 4 J. Mikulicza-Radeckiego Street, 50345 Wroclaw, Poland; kaja.tusiewicz@student.umw.edu.pl (K.T.); pawel.szpot@umw.edu.pl (P.S.); 2 Institute of Toxicology Research, 45 Kasztanowa Street, 55093 Borowa, Poland; patryk.kuropka@gmail.com (P.K.); elzbieta.workiewicz@gmail.com (E.W.);; 3 Faculty of Medicine, Wroclaw University of Science and Technology, 27 Wybrzeże Wyspiańskiego Street, 50370 Wroclaw, Poland

**Keywords:** nitrates intoxication, nitrites intoxication, epidemiology, old poisons, nitrates detection

## Abstract

In recent times, there has been a concerning and noteworthy rise in the global use of sodium nitrite for suicidal purposes. This is facilitated either through the employment of specialized “suicide kits” or by acquiring sodium nitrite through alternative means. Additionally, another occurrence contributing to nitrite poisoning is the recreational utilization of nitrites in the form of volatile aliphatic esters of nitrous acid, commonly referred to as “poppers”. Based on current available papers and reports on the subject of nitrates, nitrites, and poppers intoxications, an epidemiological analysis and evaluation of analytical methods were performed. A total of 128 papers, documenting a collective count of 492 intoxication cases, were identified. Additionally, in order to complete the epidemiological profile of nitrite poisoning, the authors briefly examined six cases of nitrite intoxication that were under investigation in our laboratory. Furthermore, a review of nitrite poisoning cases over the past 100 years shows that the old poison is still in use and poses a substantial risk to society.

## 1. Introduction

The World Health Organization reported in 2019 that more than 700,000 people die from suicide every year [1], accounting for about 1% of all causes of death worldwide. A particularly vulnerable group are young people (15–29 years old) for whom suicide is the fourth most common cause of death [1]. Searching for and being inspired by suicide methods described on the Internet, in social media, and on the darknet is a growing and horrifying phenomenon [2,3,4,5]. In recent years, reports of fatal cases of toxic substances use for suicidal purposes have been rising worldwide, with the Internet contributing significantly to the prevalence. Unintentional fatalities after new psychoactive substances ingestion are well known nowadays [6,7,8,9,10], however, an increasing number of cases of suicide attempts using old, well-known drugs and poisons such as barbiturates [11,12,13,14], cyanide [15,16,17], chloroform [18,19], azide [20,21,22,23], and DMSO [24] (which were ordered online) are reported in the literature as well. The use of such substances is facilitated by the fact that such toxic substances can be easily purchased on websites such as eBay^®^ (https://www.ebay.com/, accessed on 2 August 2023) and Amazon^®^ (https://www.amazon.com/,accessed on 2 August 2023) [24,25,26].

Most recently, a new alarming trend and a significant increase in the use of sodium nitrite for suicide purposes has been observed around the world [27,28,29,30]. Cases of fatal poisonings have already been reported in countries such as the United States [27,31,32,33], Canada [24], South Korea [34,35,36], Japan [37] Australia [38,39], Portugal [30], Spain [40], Poland [41], France [42], and Italy [43,44]. There are websites providing step-by-step instructions on how to commit suicide using sodium nitrite at home, further encouraging and describing this method as ideal due to its simplicity, quickness, and painlessness [45,46]. The origins of promoting the use of sodium nitrite as a method for suicide can be traced back to *The Peaceful Pill Handbook*, which recommends this method as simple, effective, peaceful, and painless [47]. Suicides occur with the use of “suicide kits” or with sodium nitrite purchased in other ways. So-called “suicide kits” sold on the Internet contain the appropriate amount of substance, as well as all the utensils necessary to commit suicide including instructions of how to use them [46,48]. There are also recommendations to take antiemetic and antacid drugs simultaneously with sodium nitrite ingestion to prevent vomiting, facilitate swallowing, and increase the absorption of sodium nitrite [24,49]. The most frequently detected drug in nitrite poisoning cases is metoclopramide [38,44,46,48], but also other agents such as ranitidine [38,46], ondansetron, olanzapine [30], famotidine, and cimetidine [31] have been reported. Besides nitrite poisonings, intoxication with nitrates is also possible with similar effects.

Between 2020 and 2022, six fatal cases with suspected nitrate poisoning were sent to our laboratory for toxicological analysis. Among these cases, four involved males and two involved females. Their ages ranged from 22 to 29 years old. In four cases, packaging labeled “sodium nitrite” or bottles containing a liquid with a later confirmed presence of sodium nitrite were found near the deceased. Methemoglobin levels were measured in all cases and ranged from 27% to 97.5%. All six cases were classified as suicides. The abovementioned unusual frequency of nitrite intoxications (which has been not observed in our laboratory ever before) was the basis of evaluation of this interesting phenomenon and conceptualization that was presented in this paper review.

Nitrates are present naturally in the environment and in food, especially in plants, they are introduced into the environment in the form of fertilizers, and are also used as a food additive in products such as meats and cheeses [50,51]. Sodium nitrate is considerably less toxic than sodium nitrite, however, approximately 5% of ingested nitrate is reduced to nitrite by bacteria present in the saliva and gastrointestinal tract, making it possible to develop methemoglobinemia when large amounts of nitrate are ingested [51,52]. Nitrate poisoning is not very common among adults; however, infants are particularly vulnerable to poisoning and there are known cases where poisoning has occurred as a result of preparing infant formula with contaminated well water or as a result of eating a meal containing nitrate-rich plants [50,51,53].

Another phenomenon that can lead to nitrite poisoning is the recreational use of nitrites in the form of volatile aliphatic esters of nitrous acid known as “poppers” [54]. Alkyl nitrites when inhaled provide a short-lived euphoric effect and also cause the relaxation of smooth muscles [55]. However, poppers as a source of nitrites can cause methemoglobinemia, especially if accidentally ingested instead of being inhaled. Consequently, cases of “poppers” poisoning including fatal ones have been reported [56,57].

**Figure 1:**
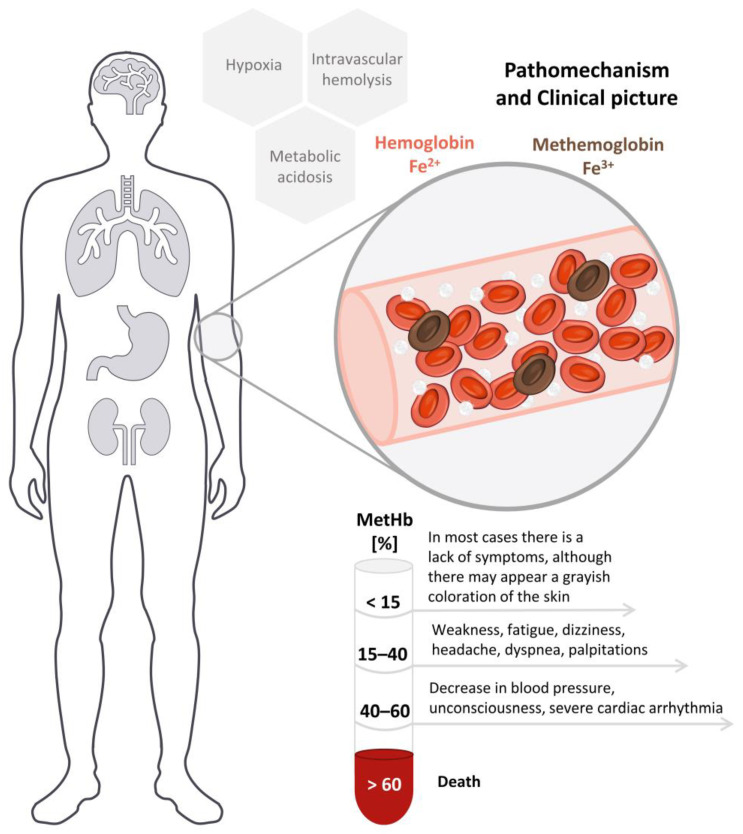
Pathomechanism and clinical picture of nitrites intoxication.

Nitrites are methemoglobin-forming compounds meaning that they cause the oxidation of the hemoglobin Fe ion from the 2nd to the 3rd oxidation state. Nitrite is most often administered by inhalation (in the form of alkyl nitrites) or the oral route, after which it is almost completely metabolized to nitrate, which is eliminated in the urine [58,59]. Three processes can be distinguished in the mechanism of poisoning: hypoxia, metabolic acidosis, and intravascular hemolysis. The change in the degree of oxidation state of Fe in hemoglobin increases the strength of its binding to oxygen and hinders the delivery of oxygen to the tissues, leading to hypoxia. In hypoxic tissues, lactic acid is formed causing the development of metabolic acidosis. Intravascular hemolysis occurs as a result of the accumulation of Heinz bodies in the erythrocytes and oxidative damage to their membranes. With methemoglobin levels above 60%, death of the poisoned person is assumed to occur mainly due to hypoxia. In the post-mortem examination, a brown color of the blood, livor mortis, and organs is characteristic in the case of a high concentration of methemoglobin [58]. The graphical representation of the pathomechanism of nitrite intoxication and the clinical picture is depicted in the Figure 1.

The aim of this work is to highlight the problem of the growing trend of the use of sodium nitrite purchased online for suicidal purposes. The authors have conducted an epidemiological study based on papers dating back to 1920 to examine the severity, changes in as well as the causes of nitrate and nitrite poisonings. A review of cases from the last 100 years has been created and, moreover, the evaluation of analytical methods used in nitrates and nitrites detection was additionally performed. What is more, analytical challenges in nitrite poisoning determination were identified. Other published review papers on this topic [28,38,60] analyze nitrite intoxication cases within a time interval of up to 20 years and do not include a detailed discussion of nitrite determination methods, so for these reasons the challenges of this determination are not pointed up. This work also provides a much more extensive meta-analysis of the poisoning problem than is present in other articles.

## 2. Epidemiology

**Figure 2:**
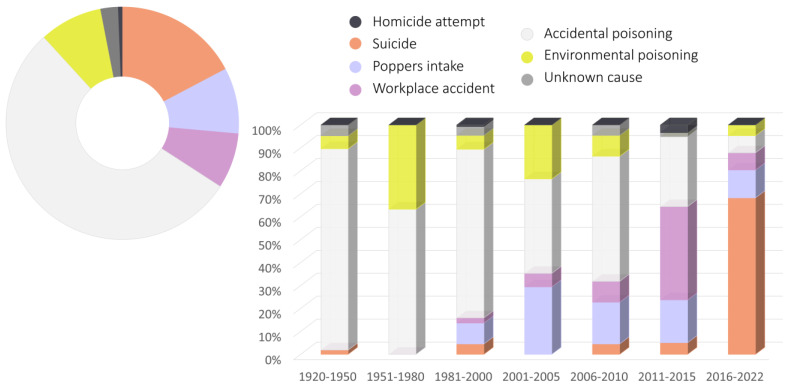
Intoxications divided by the circumstances.

**Figure 3:**
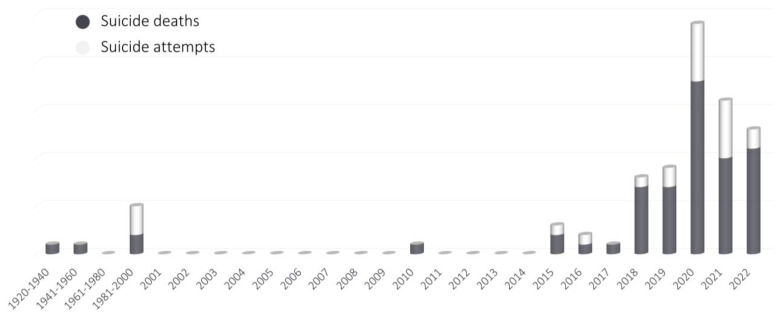
Number of suicide deaths and attempts in each year (1920–2022).

**Figure 4:**
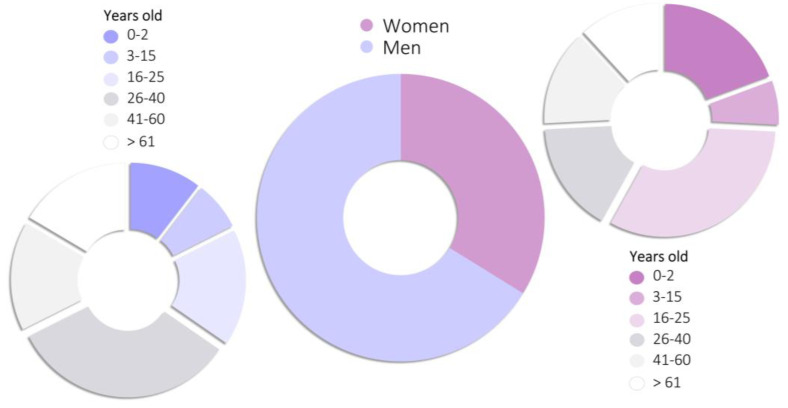
Sex of intoxicated victims.

**Figure 5:**
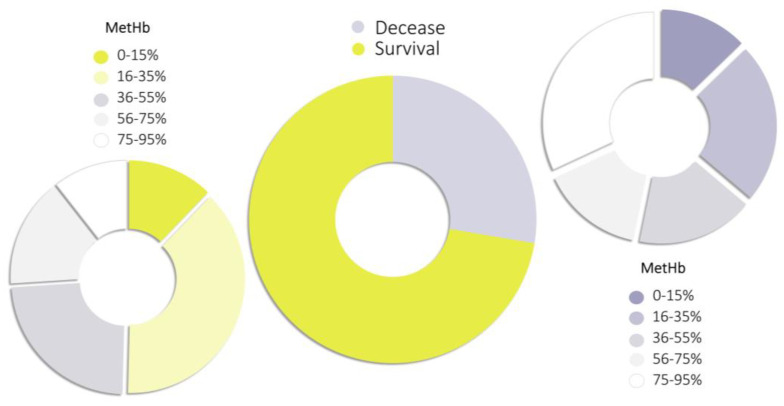
Percentage of methemoglobin (MetHb) determined in fatal and non-fatal poisonings.

**Figure 6:**
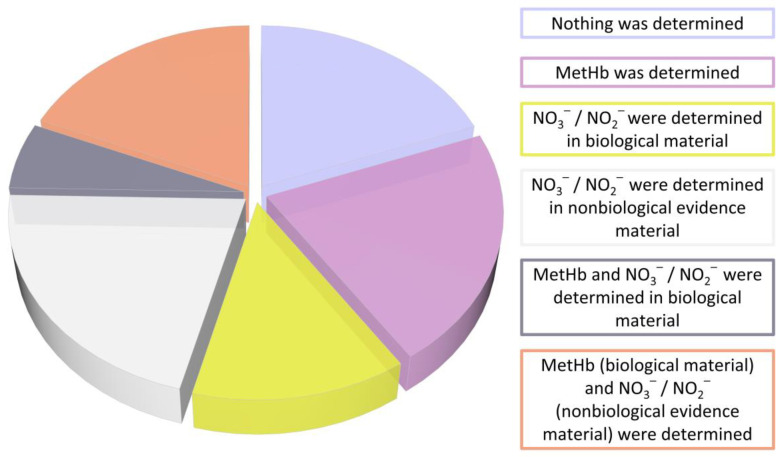
Toxicological examinations performed in intoxication cases.

Based on currently available papers and reports regarding nitrates, nitrites, and poppers intoxications, an epidemiological analysis was conducted with the use of Google Scholar and the PubMed database. The following phrases were included: “nitrate intoxication”, “nitrite intoxication”, “poppers intoxication”, “nitrate poisoning”, “nitrite poisoning”, and “poppers poisoning”. In addition, references from the literature found in search engines were searched for any omitted significant articles. A total of 128 individual papers [24,29,30,31,32,34,35,36,37,39,40,41,42,43,44,45,46,48,49,53,56,57,60,61,62,63,64,65,66,67,68,69,70,71,72,73,74,75,76,77,78,79,80,81,82,83,84,85,86,87,88,89,90,91,92,93,94,95,96,97,98,99,100,101,102,103,104,105,106,107,108,109,110,111,112,113,114,115,116,117,118,119,120,121,122,123,124,125,126,127,128,129,130,131,132,133,134,135,136,137,138,139,140,141,142,143,144,145,146,147,148,149,150,151,152,153,154,155,156,157,158,159,160,161,162,163,164,165,166] describing in total 492 cases of intoxication were found. Analyzed cases were divided into groups according to the cause of intoxication, and how the causes of poisoning changed over the years was studied (Figure 2). Moreover, the number of suicidal intoxications (Figure 3), as well as the sex and age of poisoned people (Figure 4) were studied. Another aspect that was taken into consideration was a type of a conducted toxicological analysis in order to recognize intoxication (Figure 5), and also the level of methemoglobin that was determined in fatal and non-fatal intoxications (Figure 6).

Accidental poisoning comprises almost 54% of nitrates and nitrites intoxications. The second most frequent cause of poisoning is suicide—17% of all studied cases. Poppers intake, workplace accidents, as well as environmental cases represent a similar percentage of all intoxications, i.e., 9% for each cause. In 2% of cases, the cause of intoxication was unknown. Three deliberate poisoning cases (attempted homicide) were also noted.

What is worth mentioning is the fact that causes of intoxication have changed significantly over one hundred years (Figure 2). Until 1980 intoxications comprised mainly accidental and environmental ones. One of the most frequent causes of accidental poisoning was a mistaken usage of sodium nitrite to prepare food instead of kitchen salt or sugar. The origin of sodium nitrite was usually twofold: a non-labeled reagent taken from a workplace that was mistaken for kitchen salt, or the careless and inappropriate use of too much sodium nitrite as a saltpeter [61,62,63,64,65,66,67,68,69,70]. These types of intoxications have still been present in recent years, for example, in 2018 three people were poisoned (one of them fatally) after consumption of a homemade sausage. The meal was delivered by a butcher who had used sodium nitrite as a preservative in a 30-fold higher concentration than permissible [71]. People who do not deal with meat treatment professionally can also have access to sodium nitrite, as it is used as a food additive. In 2016 a man used a 20-fold higher concentration of sodium nitrite than allowable while making homemade beef jerky. As a result, he and his daughter were hospitalized due to methemoglobinemia [72]. In 2013 a mass poisoning took place on a ship with one fatal case. As a result of a chef’s mistake, a spoon of sodium nitrite instead of kitchen salt was added to the meal for all people present on the cruise [73]. As an example of nitrate intoxication, a case of a 62-year-old man should be mentioned, who consumed an antifreeze that contained mainly ethylene glycol, but also 0.27% nitrite and 0.08% nitrate added as anticorrosion agents. The poisoning resulted in the development of methemoglobinemia [74]. Other nitrates salts are also harmful, for example, ammonium nitrate can be found in cooling compresses. A case of an intoxication being the result of drinking such formulation in order to cool body temperature was found in literature [75].

Another cause of intoxication still present over the years is environmental poisoning. Those cases involve mainly infants, who, to the age of 4–6 months, are very sensitive to methemoglobinemia developed as a result of nitrates and nitrites exposure [167]. Described cases were a result of a well water contamination [76,77,78] or the consumption of vegetables containing high concentrations of nitrates that can be reduced to nitrites by bacteria during improper storage [79,80,81,82,83,84]. Intoxications can also be atypical and observed in adults. For example, as a result of a consumption of pickled vegetables, a nitrite-induced acute kidney injury was developed [85]. It is worth mentioning that in cases where well water or vegetables are consumed, pesticide (i.e., fungicide, herbicide, or molluscicide) exposure may occur. Again, children under 3 years of age are especially more vulnerable [168,169,170,171].

At the turn of the 1970s and 1980s a new cause of poisoning occurred and can be observed to this day [54,55]. Poppers poisoning was most commonly the result of mistakenly drinking this product instead of the standard inhalation route [87,88,89,90,91,92], however, it is noteworthy that methemoglobinemia as a result of poppers inhalation has also been reported [56,93,94,95], including rare cases of intoxication with a fatal outcome [96,97]. In addition, a report of a combined non-fatal poisoning with a bupropion and alkyl nitrite overdose [98] and a single case of an unusual intravenous abuse [99] have also been reported.

Two published-to-date papers related to the use of nitrite for criminal purposes can also be found in the literature. The first one involved a daughter-in-law intentionally adding sodium nitrite into bamboo soup to poison her parents-in-law [100]. The other case refers to a series of poisonings at the end of the 20th century in Japan, where adding isobutyl nitrite to a canned coffee drink was reported [101].

Several cases among occupational intoxications are due to the industrial use of nitrates and nitrites. Between 2010 and 2012, the inadequate labelling of packaging led to a series of accidents of unintentional ingestion of antifreeze mixtures containing sodium nitrite at the construction sites in Korea [102]. Another incident occurred in 2019 in a chemical plant during methyl nitrite synthesis resulting in four intoxications due to inhalation, among which three were fatal [103].

The most alarming trend in the causes of poisoning in recent years is the greatly increased number of suicide poisonings (Figure 3). The first reported suicide using sodium nitrite purchased intentionally for this purpose occurred in 2010 [104]. Single cases of poisoning were also reported between 2015 and 2017 [105], including non-fatal cases [37,106]. Since 2018, the number of poisonings worldwide has increased significantly every year, most likely due to the recommendation by suicide-themed websites and forums for the use of sodium nitrite as an effective method of suicide [24,27,28,29,30,31,32,38,41,45,46,48,107]. Such poisonings are becoming increasingly common and have a high mortality rate.

In terms of gender, most poisoning cases involve males, and among them the most numerous age group was 26–40 years old (Figure 4). The most numerous age group among females was 16–25 years old, and the most common cause of poisoning among females of this age was suicide. The second significantly exposed group were infants (0–2 years old), which is due to their naturally greater susceptibility to nitrates and nitrites. Children (3–15 years old) are much less vulnerable and were the smallest age group among those poisoned.

More than 72% of poisoning cases ended with a non-fatal outcome, with the most commonly determined methemoglobin (MetHb) levels ranging from 16 to 35% (Figure 5). However, there are cases of non-fatal poisoning with a MetHb level greater than 75%, for example, after an unsuccessful suicide attempt a MetHb level of 92.7% was determined [108]. Such cases of survival were due to the rapid implementation of medical treatment with the use of methylene blue as an antidote. On the other hand, there are also known cases of fatal poisoning, where the measured MetHb level was less than 15%, however, this was associated with comorbidities [71]. In fatal cases, the most commonly measured level of MetHb was in the range of 75–95%, but slightly less frequently the measured level of MetHb was in the much lower range of 16–35%.

Determination of the MetHb level is a standard practice in cases of nitrate and nitrite intoxication, but it has been measured in only less than half of the described cases (Figure 5). In only about 4% of cases, MetHb measurement with the simultaneous determination of nitrate and/or nitrite in biological evidence specimens was used to confirm poisoning, and in less than 20% of cases both the MetHb measurement and determination of nitrate and/or nitrite in non-biological evidence were used to determine intoxication. In more than half of the cases, MetHb values were not measured and nitrate and/or nitrite determinations in biological evidence material (12%) or non-biological evidence (23%) were used to establish poisoning. Up to one in five cases, neither MetHb concentration testing nor nitrate and/or nitrite determination were used at all to determine the cause of poisoning. In more than 30 cases mentioned in the literature the nitrate or nitrite level was measured in gastric content and in some others in the vitreous humor. This shows the necessity to extend the application of methods used for toxic substances analysis to non-routine biological materials [172,173,174,175,176,177]. In summary, nitrates and/or nitrites were determined in biological material in only about 15% of cases, which illustrates and indicates a major analytical problem and the lack of availability of adequate reliable methods in this area. A table compiling information regarding the measured concentrations of nitrates and nitrites, analytical methods, and methemoglobin concentrations that are described in the literature can be found in the Supplementary Material Table S1.

## 3. Analytical Methods for the Determination of Nitrite and Nitrate in Biological Material

**Figure 7:**
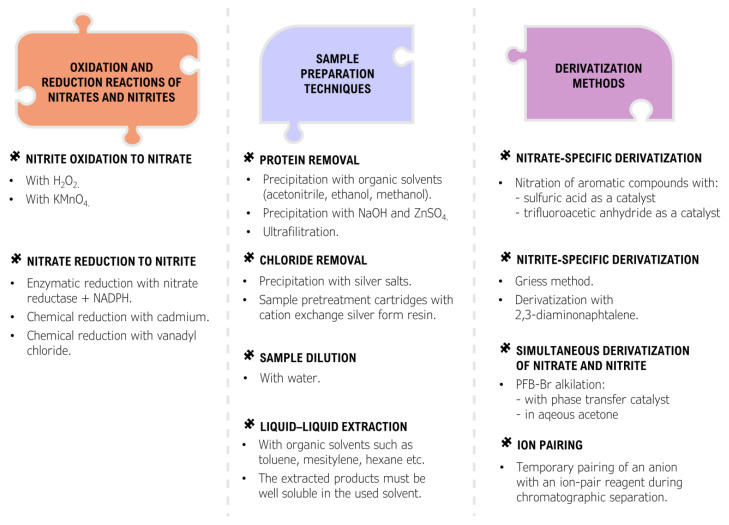
Preanalytical steps in determination of nitrates and nitrites in biological samples.

**Figure 8:**
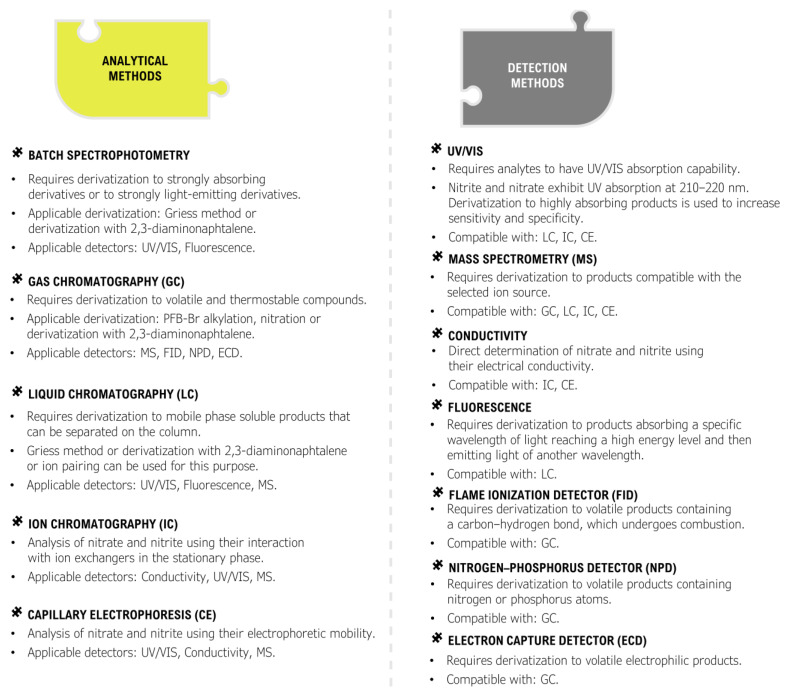
Summarization of analytical methods and detectors commonly used in nitrate and nitrite determination.

With the increasing and alarming number of suicide poisonings, there is a great demand for available, efficient, and reliable methods for the determination of nitrite in biological material. Due to the rapid conversion of nitrite to nitrate in blood, it is recommended to determine the nitrate anion as well. Many different analytical methods have been developed for the determination of nitrate and nitrite, but only a fraction of them can be used for the determination of these anions in biological material [178,179,180,181] (Figure 7 and Figure 8).

**Figure 9:**
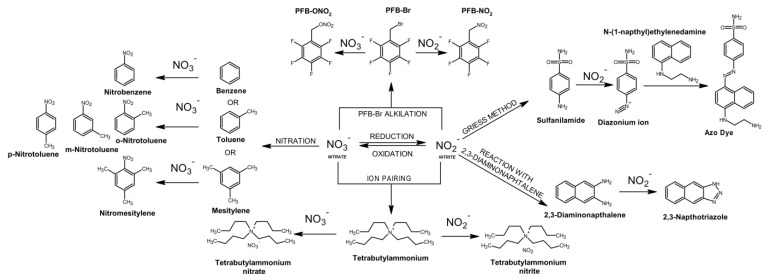
Summarization of derivatization techniques.

Even fewer analytical methods have been tested and validated for use in the analysis of complex postmortem matrices [106]. Most of the commonly used analytical techniques are unable to directly determine anions in biological material, with the exception of techniques such as ion chromatography and capillary electrophoresis [182,183,184,185]. The vast majority of the analytical techniques commonly used in forensic toxicology such as batch spectrophotometry, gas chromatography, and liquid chromatography require an appropriate derivatization procedure to create organic products that can be detected [186,187,188,189,190,191]. Many of these procedures can be problematic as they require appropriate sample preparation, can be costly and time-consuming, and are prone to a number of interferences mainly from endogenous substances present in the sample, while the analysis requires advanced instrumentation. An important aspect of the determination of nitrate and nitrite is that some of the derivatization reactions are specific only to nitrate or only to nitrite (Figure 9).

**Figure 10:**
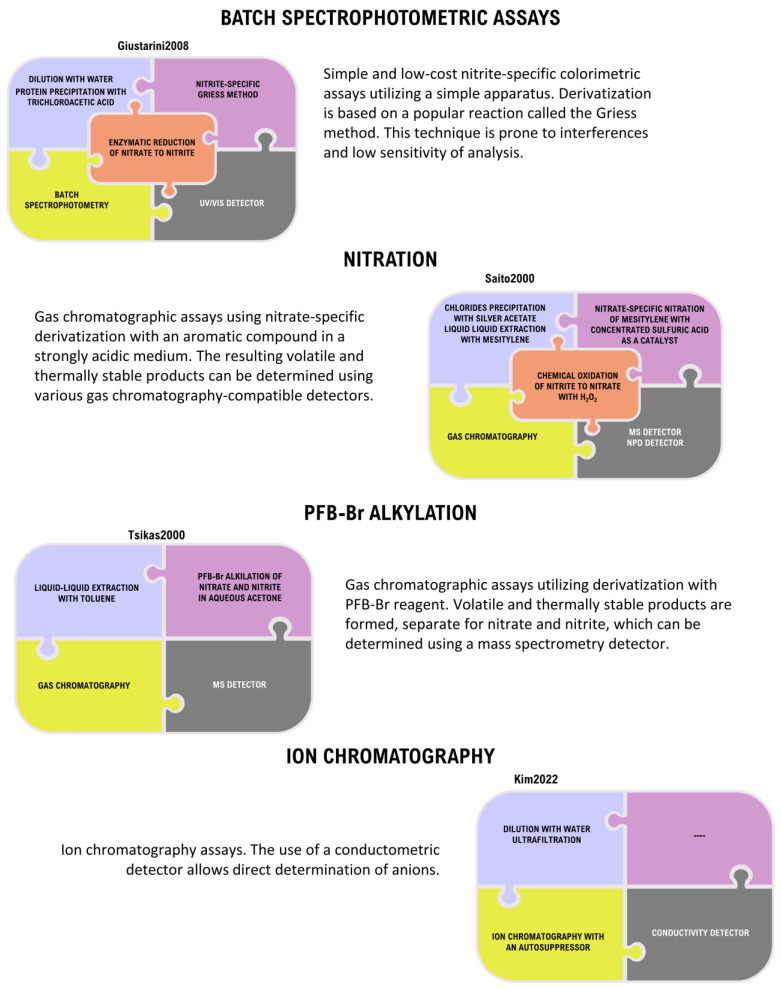
Examples of methodological approaches applied in nitrate/nitrite determination (more methods can be found in Supplementary Material Figure S1).

As a result, only one of the anions can be determined directly by a given method and the remaining anion must be determined by another method or by transforming it into the anion that can be derivatized. This approach is common and utilizes the fact that both nitrate and nitrite are readily interconvertible into each other by employing an oxidation reaction to convert nitrite to nitrate or a reduction reaction to convert nitrate to nitrite. The approaches used for the analysis of nitrate and nitrite in biological material, including both appropriate sample preparation, derivatization techniques, and analytical methods, are summarized in Figure 7 and Figure 8. In addition, specific methods developed for the analysis of nitrate and/or nitrite in biological material using various approaches are summarized in Figure 10.

Recently, the methods used in the analysis of nitrate and nitrite in biological material in cases of sodium nitrite poisoning are mainly ion chromatography [159] and spectrophotometric analysis based on the Griess method [30,41]. However, the inexpensive and relatively easily accessible Griess method has a lot of drawbacks and limitations. Griess colorimetric assay is one of the oldest and best-recognized methods for the determination of nitrate and nitrite [191] (Figure 10). Over the years, this reaction has been refined and has many alternative versions, and the most common today involves the diazotization reaction of nitrite with sulfanilamide and subsequent coupling with N-(1-naphthyl)ethylenediamine (Figure 9). The resulting azo compound absorbs radiation in the visible range at about 540 nm [191]. The batch spectrophotometric approach is a simple and easily achievable technique, and the required derivatization reagents can be purchased in ready-to-use kits made for these purposes [191,192,193]. However, this technique has relatively low sensitivity, and success in obtaining accurate results depends on appropriate sample purification. Complex biological matrices contain many compounds that can cause turbidity in the sample but also absorption and interference with detection. In particular, hemoglobin in hemolyzed blood intensely absorbs radiation in the 540 nm range [191,194]. In addition, many endogenous compounds can adversely affect the derivatization reaction causing the risk of false negative results [191,193,194]. The Batch Griess assay is one of the fastest, simplest, and cheapest methods for the determination of nitrate and nitrite and is therefore often chosen for the determination of these anions in cases of fatal poisoning [105]. However, this method often gives different results compared to techniques considered to be more reliable and referential (for example, those based on mass spectrometry), so it should be used with caution and more reliable techniques should be used whenever possible [191,195].

A much more promising method is ion chromatography, which does not require the use of problematic reduction and derivatization processes. IC is characterized by the ability to analyze multiple anions simultaneously in both simple and complex matrices and thus it has been frequently used for the analysis of nitrates and nitrites in poisoning cases [159] (Figure 10). Unfortunately, ion chromatography is not well established and available in all forensic toxicology laboratories.

In forensic toxicology, but also in other related subfields such as entomotoxicology or veterinary forensic toxicology, mass spectrometry remains the gold standard and the most widespread analytical technique for the determination of toxic substances [196,197,198,199,200,201,202]. A great advantage of mass spectrometry methods is the possibility to perform quantitative measurements using internal standards in the form of isotopically labeled analogs of the substances of interest. The use of commercially available ^15^NO_3_^−^ and ^15^NO_2_^−^ salts as internal standards added to biological material simplifies the analytical process and provides reliable results, as these standards undergo identical transformations and processes (extraction, derivatization, reduction, chromatographic separation) during the whole analytical process as naturally occurring nitrates and nitrites [203,204]. These anions cannot be analyzed directly by GC–MS, but there are two main derivatization techniques that provide thermally stable and volatile products that can be analyzed with this method: nitration and alkylation with pentafluorobenzyl bromide (PFB-Br).

The principle of the nitration method is the nitrate-specific reaction of an aromatic compound with a nitrate anion under acidic conditions [203]. A major drawback of the nitration method is the use of concentrated sulfuric acid as a catalyst for the reaction. There are studies indicating that the use of sulfuric acid causes a positive interference in the determination of nitrate, due to the decomposition and release of nitrate from endogenous compounds present in the plasma, such as various nitroso compounds [203]. Moreover, working with concentrated sulfuric acid itself requires care and caution due to the release of large amounts of heat during its addition to the aqueous phase. The high levels of chlorides found endogenously in the sample make it impossible to perform derivatization, thus they must be removed by chemical precipitation or through special cartridges before adding sulfuric acid. Similarly, the presence of proteins makes derivatization difficult, hence the need to precipitate them or remove them with ultrafiltration [203,205].

The basis of the second derivatization method is the alkylation reaction with the derivatizing reagent pentafluorobenzyl bromide (PFB-Br) (Figure 8). This reagent reacts with both nitrate and nitrite to form the nitric acid ester PFB-ONO_2_ and the nitro derivative PFB-NO_2_, respectively. The resulting products are volatile, and the presence of fluorine atoms results in strong electron-capturing properties that enhance the detection and greatly increase the sensitivity of the analysis based on GC–MS [204]. A major advantage of alkylation with PFB-Br is the simultaneous single-step derivatization reaction for both ions, thus not requiring the problematic reduction or oxidation reaction of one anion into the other. The reaction rates for both anions are different and the simultaneous derivatization requires the optimization of conditions including temperature, reaction time, and pH [188,203]. More details regarding other approaches can be found in the Supplementary Material (analysis was extended with other methodological papers [206,207,208,209,210,211,212,213,214,215,216,217,218,219,220,221]).

## 4. Conclusions

The phenomenon of sodium nitrite suicide poisoning is a growing and alarming new trend. An increasing number of countries are reporting more cases of fatal poisonings, and it is possible that many more remain unrecorded. The reason for the prevalence of this method of suicide can be attributed to online sources. The situation is exacerbated by the relative ease of availability of the substance. Thus, the availability of this substance, especially in suicidal quantities, should be limited and controlled. There is a widespread debate about restricting the sale of these type of substances and some websites are recalling products such as sodium nitrite [222]. The UK has listed sodium nitrite as a “notifiable substance”, which means that sellers have to report suspicious purchases to the authorities. However, it is unclear whether such measures will reduce deliberate poisonings [195]. Furthermore, a review of nitrite poisoning cases over the past 100 years shows that the old poison is still in use and poses a significant threat to society.

The postmortem measurement of MetHb concentration appears to be the standard approach in cases of sodium nitrite poisoning. However, determining the cause of poisoning solely on the basis of methemoglobinemia is not recommended, as this condition can also be caused by many other substances, and the determination of MetHb itself can be problematic. In addition, MetHb concentrations reported in fatal poisoning cases occur over a wide range, often not exceeding the minimum 60% reference lethal saturation described in the literature (for more information see Table S1 in the Supplementary Material). Therefore, when determining death from nitrite poisoning, the determination of anions in biological material is recommended, and other factors, such as the description of the scene, the presence of a suicide note, and substances secured at the scene, should also be carefully considered. The determination of nitrite in blood can often be impossible due to its instability in this material. However, it can often be detected in alternative materials such as gastric contents, pericardial fluid, or the vitreous humor where it is not exposed to oxidizing agents, especially red blood cell components. Nevertheless, the negative result of the nitrite presence in these materials cannot exclude sodium nitrite poisoning and an elevated concentration of nitrate in the blood should also be considered as an indicator of poisoning.

There are a number of methods for the determination of nitrite and nitrate, but most of them cannot be or are difficult to use for complex matrices. In addition, although a variety of methods are used in clinical cases, only a small number of methods have been performed and validated for forensically relevant postmortem material. All this makes it necessary to develop techniques that are accessible, relatively inexpensive, and reliable. The cheapest, widely available, and low-cost Griess assay method is often used to determine nitrite in fatal poisonings, but this method is prone to interference, often providing false results. More reliable methods are definitely those using isotopic labeled standards, but these techniques require a complicated sample preparation and derivatization procedure as well as sophisticated instrumentation. Alternatives to these techniques can be ion chromatography and capillary electrophoresis, which do not require a derivatization step. However, these techniques can also have difficulties related to sample preparation, applicability to complex matrices, selectivity, and sensitivity, and are not yet widely used and accessible in forensic toxicology. Future research should focus not only on developing reliable analytical methods capable of determining nitrite in complex biological materials, but also on finding indicators and biomarkers that will enable the unambiguous identification of sodium nitrite poisoning.

## Data Availability

Not applicable.

## Supplementary Materials

The following supporting information can be downloaded at: https://www.mdpi.com/article/10.3390/toxics11100832/s1, Figure S1: details regarding other approaches to nitrites and nitrates determination in biological material; Table S1: Measured concentrations of nitrite and/or nitrate in biological material in fatal poisoning cases.

## References

[1] World Health Organization (2021). Suicide Worldwide in 2019: Global Health Estimates.

[2] Marchant A., Hawton K., Stewart A., Montgomery P., Singaravelu V., Lloyd K., Purdy N., Daine K., John A. (2017). A Systematic Review of the Relationship between Internet Use, Self-Harm and Suicidal Behaviour in Young People: The Good, the Bad and the Unknown. PLoS ONE.

[3] Biddle L., Gunnell D., Owen-Smith A., Potokar J., Longson D., Hawton K., Kapur N., Donovan J. (2012). Information Sources Used by the Suicidal to Inform Choice of Method. J. Affect. Disord..

[4] Mörch C.M., Côté L.P., Corthésy-Blondin L., Plourde-Léveillé L., Dargis L., Mishara B.L. (2018). The Darknet and Suicide. J. Affect. Disord..

[5] Lopez-Castroman J., Moulahi B., Azé J., Bringay S., Deninotti J., Guillaume S., Baca-Garcia E. (2020). Mining Social Networks to Improve Suicide Prevention: A Scoping Review. J. Neurosci. Res..

[6] UNODC (2022). Current NPS Treats. Volume V. https://www.unodc.org/documents/scientific/Current_NPS_Threats_V.pdf.

[7] Nowak K., Szpot P., Zawadzki M. (2021). Fatal intoxication with U-47700 in combination with other NPS (*N*-ethylhexedrone, adinazolam, 4-CIC, 4-CMC) confirmed by identification and quantification in autopsy specimens and evidences. Forensic Toxicol..

[8] Zawadzki M., Wachełko O., Tusiewicz K., Szpot P. (2022). Severe poisoning after smoking a mixture of 4-fluoroisobutyryl fentanyl (4-FiBF) and alpha-pyrolidinoisohexaphenone (α-PiHP). J. Forensic Leg. Med..

[9] Zawadzki M., Chłopaś-Konowałek A., Nowak K., Wachełko O., Szpot P. (2021). Quantification of 5F-CUMYL-P7AICA in blood and urine from an authentic fatality associated with its consumption by UHPLC–MS/MS. Forensic Toxicol..

[10] Tusiewicz K., Chłopaś-Konowałek A., Wachełko O., Zawadzki M., Szpot P. (2023). A fatal case involving the highest ever reported 4-CMC concentration. J. Forensic Sci..

[11] Stephenson L., Kenneally M., van den Heuvel C., Humphries M., Stockham P., Byard R.W. (2021). Recent Trends in Barbiturate Detection in Medicolegal Deaths. Leg. Med..

[12] Solbeck P., Snowdon V., Rajagopalan A., Jhirad R. (2019). Suicide by Fatal Pentobarbital Intoxication in Ontario, Canada, from 2012 to 2015. J. Forensic Sci..

[13] Campbell G., Darke S., Zahra E., Duflou J., Shand F., Lappin J. (2021). Trends and Characteristics in Barbiturate Deaths Australia 2000-2019: A National Retrospective Study. Clin. Toxicol.

[14] van den Hondel K.E., Punt P., Dorn T., Ceelen M., Reijnders U. (2020). The Rise of Suicides Using a Deadly Dose of Barbiturates in Amsterdam and Rotterdam, the Netherlands, between 2006 and 2017. J. Forensic Leg. Med..

[15] Tournel G., le Garff E., Humbert L., Wiart J.-F., Garat A., Hedouin V., Allorge D. (2014). O41: Dark Web Shopping: A Case Report of a Cyanide Suicide. Toxicol. Anal. Et. Clin..

[16] le Garff E., Delannoy Y., Mesli V., Allorge D., Hédouin V., Tournel G. (2016). Cyanide Suicide after Deep Web Shopping: A Case Report. Am. J. Forensic Med. Pathol..

[17] Wachełko O., Chłopaś-Konowałek A., Zawadzki M., Szpot P. (2022). Old Poison, New Problem: Cyanide Fatal Intoxications As-sociated with Internet Shopping. J. Anal. Toxicol..

[18] Zorro A.R. (2014). Asphyxial Suicide by Inhalation of Chloroform inside a Plastic Bag. J. Forensic Leg. Med..

[19] Tusiewicz K., Wachełko O., Zawadzki M., Chłopaś-Konowałek A., Jurek T., Kawecki J., Szpot P. (2022). The Dark Side of Social Media: Two Deaths Related with Chloroform Intoxication. J. Forensic Sci..

[20] Tat J., Heskett K., Satomi S., Pilz R.B., Golomb B.A., Boss G.R. (2021). Sodium Azide Poisoning: A Narrative Review. Clin. Toxicol..

[21] Rojek S., Hydzik P., Gomółka E., Kula K., Kłys M. (2015). Clinical and Analytical Problems of Sodium Azide Poisonings as Exem-plified by a Case of Fatal Suicidal Poisoning. Arch. Med. Sadowej Kryminol..

[22] Wachełko O., Zawadzki M., Szpot P. (2021). A novel procedure for stabilization of azide in biological samples and method for its determination (HS-GC-FID/FID). Sci. Rep..

[23] Ciesla M.M., Calello D.P., Nelson L.S. (2018). When the Poisoned Risk Poisoning Others: Fatal Sodium Azide Overdose. Emerg Med..

[24] Hickey T.B.M., MacNeil J.A., Hansmeyer C., Pickup M.J. (2021). Fatal Methemoglobinemia: A Case Series Highlighting a New Trend in Intentional Sodium Nitrite or Sodium Nitrate Ingestion as a Method of Suicide. Forensic Sci. Int..

[25] Cantrell F.L. (2005). Look What I Found! Poison Hunting on EBay^®^. Clin. Toxicol..

[26] Leonard J.B., Hines E.Q., Anderson B.D. (2020). Prime Eligible Poisons: Identification of Extremely Hazardous Substances Available on Amazon.Com^®^. Clin. Toxicol..

[27] McCann S.D., Tweet M.S., Wahl M.S. (2021). Rising Incidence and High Mortality in Intentional Sodium Nitrite Exposures Reported to US Poison Centers. Clin. Toxicol..

[28] Mudan A., Lebin J.A., Smollin C.G. (2021). National Poison Data System (NPDS) Review of Intentional Sodium Nitrite Ingestions 2009–2019. Toxicol Commun.

[29] Mudan A., Repplinger D., Lebin J., Lewis J., Vohra R., Smollin C. (2020). Severe Methemoglobinemia and Death from Intentional Sodium Nitrite Ingestions. J. Emerg. Med..

[30] Durão C., Pedrosa F., Dinis-Oliveira R.J. (2021). Another Suicide by Sodium Nitrite and Multiple Drugs: An Alarming Trend for “Exit”?. Forensic Sci. Med. Pathol..

[31] Dean D.E., Looman K.B., Topmiller R.G. (2021). Fatal Methemoglobinemia in Three Suicidal Sodium Nitrite Poisonings. J. Forensic Sci..

[32] Sedhai Y.R., Atreya A., Basnyat S., Phuyal P., Pokhrel S. (2022). The Use of Sodium Nitrite for Deliberate Self-Harm, and the Online Suicide Market: Should We Care?. Med. Leg. J..

[33] Vodovar D., Megarbane B. (2022). Are Sodium Nitrite Exposures Increasing in the United States?. Clin. Toxicol..

[34] Kim M., Kim S., Yang W., Sim J. (2022). Determination of Nitrite and Nitrate in Postmortem Whole Blood Samples of 10 Sodium Nitrite Poisoning Cases: The Importance of Nitrate in Determining Nitrite Poisoning. Forensic Sci. Int..

[35] Park M.J., Kim O., Ha H. (2020). Death by Nitrite Intoxication: Report of 14 Cases. Korean J. Leg. Med..

[36] Mun S.H., Park G.J., Lee J.H., Kim Y.M., Chai H.S., Kim S.C. (2022). Two Cases of Fatal Methemoglobinemia Caused by Self-Poisoning with Sodium Nitrite: A Case Report. Medicine.

[37] Katabami K., Hayakawa M., Gando S. (2016). Severe Methemoglobinemia Due to Sodium Nitrite Poisoning. Case Rep. Emerg. Med..

[38] Stephenson L., Wills S., van den Heuvel C., Humphries M., Byard R.W. (2022). Increasing Use of Sodium Nitrite in Suicides—An Emerging Trend. Forensic Sci. Med. Pathol..

[39] Perkins C.J., Wahl G.E., Gillett M.J. (2022). A Case of Pseudohyperchloraemia Caused by Sodium Nitrate Ingestion. Clin. Toxicol..

[40] García Caballero C., González del Campo Rollán V., Martínez González M.A. (2023). Suicidal Poisoning by Sodium Nitrite: A Dangerous Mode from Internet. In Regard of a Case. Span. J. Leg. Med..

[41] Tomsia M., Głaz M., Nowicka J., Szczepański M. (2021). Sodium Nitrite Detection in Costal Cartilage and Vitreous Humor—Case Report of Fatal Poisoning with Sodium Nitrite. J. Forensic Leg. Med..

[42] Vodovar D., Tournoud C., Boltz P., Paradis C., Puskarczyk E. (2022). Severe Intentional Sodium Nitrite Poisoning Is Also Being Seen in France. Clin. Toxicol..

[43] Barranco R., Frigiolini F.M.E., Orcioni G.F., Malandrino M., Salomone A., Ventura F. (2021). A Rare Case of Fatal Self-Poisoning with Sodium Nitrite: Autopsy and Toxicological Findings. Am. J. Forensic Med. Pathol..

[44] Bugelli V., Tarozzi I., Manetti A.C., Stefanelli F., di Paolo M., Chericoni S. (2022). Four Cases of Sodium Nitrite Suicidal Ingestion: A New Trend and a Relevant Forensic Pathology and Toxicology Challenge. Leg. Med..

[45] Yoon J.C., Kim S.E. (2022). Suicide Attempt Using Sodium Nitrite Ordered on the Internet: Two Case Reports. Medicine.

[46] Durão C., Pedrosa F., Dinis-Oliveira R.J. (2020). A Fatal Case by a Suicide Kit Containing Sodium Nitrite Ordered on the Internet. J. Forensic Leg. Med..

[47] Nitschke P., Steward F. (2006). The Peaceful Pill Handbook.

[48] Matheux A., Loiseau M., Sabini S., Cavard S., Advenier A.-S., Pasquet A., Errard J.-F., Devresse A., Villain T., Gosse R. (2022). Suicide of a Young Woman Using a Kit Containing Sodium Nitrite Ordered on the Internet. Toxicol. Anal. Et. Clin..

[49] Saleh D., Lucyk S., McGillis E. (2022). Methemoglobinemia Caused by Sodium Nitrite Overdose. CMAJ.

[50] Greer F.R., Shannon M. (2005). Infant Methemoglobinemia: The Role of Dietary Nitrate in Food and Water. Pediatrics.

[51] Santamaria P. (2006). Nitrate in Vegetables: Toxicity, Content, Intake and EC Regulation. J. Sci. Food Agric..

[52] van Velzen A.G., Sips A.J.A.M., Schothorst R.C., Lambers A.C., Meulenbelt J. (2008). The Oral Bioavailability of Nitrate from Nitrate-Rich Vegetables in Humans. Toxicol. Lett..

[53] Joosen D., Stolk L., Henry R. (2014). A Non-Fatal Intoxication with a High-Dose Sodium Nitrate. BMJ Case Rep..

[54] Le A., Yockey A., Palamar J.J. (2020). Use of “Poppers” among Adults in the United States, 2015–2017. J. Psychoact. Drugs.

[55] Romanelli F., Smith K.M., Thornton A.C., Pomeroy C. (2004). Poppers: Epidemiology and Clinical Management of Inhaled Nitrite Abuse. Pharmacotherapy.

[56] Ranchon G., Mollard F., Lainé N., Malick P., Robert D. (2008). Poppers-Induced Methemoglobinemia: An Unusual Cause of Cy-anosis. Eur. J. Emerg. Med..

[57] Al-Lawati A., Murch N. (2012). Acquired Methemoglobinaemia. Sultan Qaboos Univ. Med. J..

[58] Zawadzki M., Teresiński G. (2020). Związki methemoglobinotwórcze. Medycyna Sądowa.

[59] Baselt R.C. (2017). Nitrite. Disposition of Toxic Drugs and Chemicals in Man.

[60] Andelhofs D., Van Den Bogaert W., Lepla B., Croes K., Van de Voorde W. (2023). Suicidal Sodium Nitrite Intoxication: A Case Report Focusing on the Postmortem Findings and Toxicological Analyses—Review of the Literature. Forensic Sci. Med. Pathol..

[61] Greenberg M., Birnkrant W.B., Schiftner J.J. (1945). Outbreak of Sodium Nitrite Poisoning. Am. J. Public. Health Nations Health.

[62] Mcquiston T.A.C. (1936). Fatal poisoning by sodium nitrite. Lancet.

[63] Padberg L.R. (1939). Three fatal cases of sodium nitrite poisoning. AMA.

[64] Orgeron J.D., Martin J.D., Caraway C.T., Martine R.M., Hauser G.H. (1957). Methemoglobinemia from Eating Meat With High Nitrite Content. Public. Health Rep..

[65] Walley T., Flanagan M. (1987). Nitrite-Induced Methaemoglobinaemia. Postgrad. Med. J..

[66] Gautami S., Rao R.N., Raghuram T.C., Rajagopalan S., Bhat R.v. (1995). Accidental Acute Fatal Sodium Nitrite Poisoning. Clin. Toxicol..

[67] Kennedy N., Smith C.P., McWhinney P. (1997). Faulty Sausage Production Causing Methaemoglobinaemia. Arch. Dis. Child..

[68] Finan A., Keenan P., Donovan F.O., Mayne P., Murphy J. (1998). Lesson of the Week: Methaemoglobinaemia Associated with Sodium Nitrite in Three Siblings. BMJ.

[69] Yang J.J., Lin N., Lv R., Sun J., Zhao F., Zhang J., Xu J.G. (2005). Methemoglobinemia Misdiagnosed as Ruptured Ectopic Preg-nancy. Acta Anaesthesiol. Scand..

[70] Tung S.P., How C.K., Chern C.H. (2006). Methaemoglobinaemia Secondary to the Ingestion of Sodium Nitrite in Mistake for Common Salt. Resuscitation.

[71] Cvetković D., Živković V., Lukić V., Nikolić S. (2019). Sodium Nitrite Food Poisoning in One Family. Forensic Sci. Med. Pathol..

[72] Theobald J.L., Spoelhof R., Pallasch E.M., Mycyk M.B. (2018). The Beef Jerky Blues Methemoglobinemia from Home Cured Meat. Pediatr. Emerg. Care.

[73] Lee C., Jang E.J., Yum H., Choi Y.S., Hong J. (2017). Unintentional Mass Sodium Nitrite Poisoning with a Fatality. Clin. Toxicol..

[74] Farkas A.N., Scoccimarro A., Pizon A.F. (2017). Methemoglobinemia Due to Antifreeze Ingestion. N. Engl. J. Med..

[75] Challoner K.R., Mccarron M.M. (1988). Ammonium nitrate cold pack ingestion. J. Emerg. Med..

[76] Faucett R.L., Miller H.C. (1946). Methemoglobinemia Occurring in Infants Fed Milk Diluted with Well Water of High Nitrate Content. J. Pediatr..

[77] Comly H.H. (1945). Cyanosis in Infants Caused by Nitrates in Well Water. J. Am. Med. Assoc..

[78] Ayebo A., Kross B.C., Vlad M. (1997). Infant Methemoglobinemia in the Transylvania Region of Romania. Int. J. Occup. Environ. Health.

[79] Simon C. (1966). Nitrite Poisoning from Spinach. Lancet.

[80] Chan T.Y. (1996). Food-Borne Nitrates and Nitrites as a Cause of Methemoglobinemia. Southeast Asian J. Trop. Med. Public. Health.

[81] Chan T.Y.K. (2011). Vegetable-Borne Nitrate and Nitrite and the Risk of Methaemoglobinaemia. Toxicol. Lett..

[82] Bosset A.J., Stucki P., Roback M.G., Gehri M. (2005). Severe Methemoglobinemia Due to Food Intoxication in Infants. Pediatr. Emerg. Care.

[83] Sanchez-Echaniz J., Benito-Fernández J., Mintegui-Raso S. (2001). Methemoglobinemia and Consumption of Vegetables in Infants. Pediatrics.

[84] Savino F., Maccario S., Guidi C., Castagno E., Farinasso D., Cresi F., Silvestro L., Mussa G.C. (2006). Methemoglobinemia Caused by the Ingestion of Courgette Soup given in Order to Resolve Constipation in Two Formula-Fed Infants. Ann. Nutr. Metab..

[85] Peng T., Hu Z., Yang X., Gao Y., Ma C. (2018). Nitrite-Induced Acute Kidney Injury with Secondary Hyperparathyroidism. Medicine.

[86] Shesser R., Mitchell J., Edelstein S. (1981). Methemoglobinemia from Isobutyl Nitrite Preparations. Ann. Emerg. Med..

[87] Dudley M.J., Solomon T. (1993). A Case of Methaemoglobinaemia. Arch. Emerg. Med..

[88] Edwards R.J., Ujma J. (1995). Extreme Methaemoglobinaemia Secondary to Recreational Use of Amyl Nitrite. J. Accid. Emerg. Med..

[89] Stambach T., Haire K., Soni N., Booth J. (1997). Saturday Night Blue—A Case of near Fatal Poisoning from the Abuse of Amyl Nitrite. Emerg. Med. J..

[90] Jansen T., Barnung S., Mortensen C.R., Jansen E.C. (2003). Isobutyl-Nitrite-Induced Methemoglobinemia; Treatment with an Ex-change Blood Transfusion during Hyperbaric Oxygenation Case Report. Acta Anaesthesiol. Scand..

[91] Pain S., Chavant F., Fauconneau B., Guenezan J., Marjanovic N., Lardeur J.Y., Brunet B., Perault-Pochat M.C. (2017). Dangerous intoxication after oral ingestion of poppers (alkyl nitrites): Two case reports. Therapie.

[92] Wilkerson R.G. (2010). Getting the Blues at a Rock Concert: A Case of Severe Methaemoglobinaemia. Emerg. Med. Australas..

[93] Modarai B. (2002). Methylene Blue: A Treatment for Severe Methaemoglobinaemia Secondary to Misuse of Amyl Nitrite. Emerg. Med. J..

[94] Janssens U., Hillen S., Janssens T., Grafe J. (2019). Methemoglobinemia after Inhalation of Poppers. Med. Klin. Intensiv. Notfmed.

[95] Lindenmann J., Matzi V., Kaufmann P., Krisper P., Maier A., Porubsky C., Smolle-Juettner F.M. (2006). Hyperbaric Oxygenation in the Treatment of Life-Threatening Isobutyl Nitrite-Induced Methemoglobinemia—A Case Report. Inhal. Toxicol..

[96] Machabert R., Testud F., Descotes J. (1994). Methaemoglobinaemia due to amyl nitrite inhalation: A case report. Hum. Exp. Toxicol..

[97] Bradberry S.M., Whittington R.M., Parry D.A., Allister Vale J. (1994). Fatal Methemoglobinemia Due to Inhalation of Isobutyl Nitrite. J. Toxicol. Clin. Toxicol..

[98] Batista F., Alves C., Trindade M., Duarte J.A., Marques R. (2019). Methaemoglobinemia Induced by Poppers and Bupropion In-toxication in the Emergency Department. Eur. J. Case Rep. Intern. Med..

[99] Reisinger A., Vogt S., Essl A., Rauch I., Bangerl F., Eller P., Hackl G. (2020). Lessons of the Month 3: Intravenous Poppers Abuse: Case Report, Management and Possible Complications. Clin. Med..

[100] Su Y.F., Lu L.H., Hsu T.H., Chang S.L., Lin R.T. (2012). Successful Treatment of Methemoglobinemia in an Elderly Couple with Severe Cyanosis: Two Case Reports. J. Med. Case Rep..

[101] Seto Y., Kataoka M., Tsuge K., Takaesu H. (2000). Pitfalls in the Toxicological Analysis of an Isobutyl Nitrite-Adulterated Coffee Drink. Anal. Chem..

[102] Sohn C.H., Seo D.W., Ryoo S.M., Lee J.H., Kim W.Y., Lim K.S., Oh B.J. (2014). Life-Threatening Methemoglobinemia after Unin-tentional Ingestion of Antifreeze Admixtures Containing Sodium Nitrite in the Construction Sites. Clin. Toxicol..

[103] Huang S., Wang R., Guo B., Ruan H., Ma J., Ren L., Liu L. (2020). Fatal Methemoglobinemia Due to Acute Inhalation of Methyl Nitrite in an Industrial Accident. J. Forensic Sci..

[104] Harvey M., Cave G., Chanwai G. (2010). Fatal Methaemoglobinaemia Induced by Self-Poisoning with Sodium Nitrite. Emerg. Med. Austral..

[105] Nishiguchi M., Nushida H., Okudaira N., Nishio H. (2014). An Autopsy Case of Fatal Methemoglobinemia Due to Ingestion of Sodium Nitrite. J. Forensic Sci..

[106] Yan H., Zhuo X., Shen B., Xiang P., Shen M. (2016). Determination of Nitrite in Whole Blood by High-Performance Liquid Chro-matography with Electrochemical Detection and a Case of Nitrite Poisoning. J. Forensic Sci..

[107] Pires K.D., Hart K., Tomassoni A.J. (2022). Internet-Assisted Suicide by Nitrite Poisoning—A Case Report and Increase in Reported Intentional Nitrite/Nitrate Exposures in U.S. Poison Center Data. Clin. Toxicol..

[108] dela Cruz M., Glick J., Merker S.H., Vearrier D. (2018). Survival after Severe Methemoglobinemia Secondary to Sodium Nitrate Ingestion. Toxicol. Commun..

[109] Palmer A.A. (1933). Fatal Poisoning by Sodium Nitrite. Med. J. Aust..

[110] Buch O. (1952). Massenvergiftung Durch Natriumnitrit. Slg Vergiftfalle Arch. Toxicol..

[111] Tepperman J. (1951). Methemoglobinemic Cyanosis. J. Am. Med. Assoc..

[112] Lecks H.I. (1950). Methemoglobinemia in Infancy. Am. J. Dis. Child..

[113] Oppé T.E. (1951). Methæmoglobinæmia Due to Sodium Nitrite. Lancet.

[114] Barton G.M.G. (1954). A Fatal Case of Sodium Nitrite Poisoning. Lancet.

[115] Bucklin R. (1960). Fatal Methemoglobinemia Due to Well Water Nitrates. Ann. Intern. Med..

[116] Singley T.L. (1962). Secondary Methemoglobinemia Due to the Adulteration of Fish with Sodium Nitrite. Ann. Intern. Med..

[117] Bakshi S.P., Fahey J.L., Pierce L.E. (1967). Sausage Cyanosis—Acquired Methemoglobinemic Nitrite Poisoning. N. Engl. J. Med..

[118] Harris J.C. (1979). Methemoglobinemia Resulting from Absorption of Nitrates. JAMA.

[119] Ten Brink W.A., Wiezer J.H., Luijpen A.F., Van Heijst A.N., Pikaar S.A., Seldenrijk R. (1982). Nitrate Poisoning Caused by Food Contaminated with Cooling Fluid. J. Toxicol. Clin. Toxicol..

[120] Aquanno J.J., Chan K.M., Dietzler D.N. (1981). Accidental Poisoning of Two Laboratory Technologists with Sodium Nitrite. Clin. Chem..

[121] Laaban J.P. (1985). Amyl Nitrite Poppers and Methemoglobinemia. Ann. Intern. Med..

[122] Forsyth R.J., Moulden A. (1991). Methaemoglobinaemia after Ingestion of Amyl Nitrite. Arch. Dis. Child..

[123] Askew G.L., Sosin D.M., Finelli L., Genese C.A., Sorhage F.E., Spitalny K.C. (1994). Boilerbaisse: An Outbreak of Methemoglo-binemia in New Jersey in 1992. Pediatrics.

[124] Gosnold J.K., Johnson G.S. (1993). Methaemoglobinaemia as a Result of Sodium Nitrate Poisoning. Arch. Emerg. Med..

[125] Zalstein S. (1993). Methaemoglobinaemia Due to Nitrite Poisoning. Emerg. Med..

[126] Saito T., Takeichi S., Yukawa N., Osawa M. (1996). Fatal Methemoglobinemia Caused by Liniment Solutions Containing Sodium Nitrite. J. Forensic Sci..

[127] Freeman L., Wolford R.W. (1996). Methemoglobinemia Secondary to Cleaning Solution Ingestion. J. Emerg. Med..

[128] Malhotra R., Hughes G. (1996). Methaemoglobinaemia Presenting with Status Epilepticus. J. Accid. Emerg. Med..

[129] Le Cam Y., Carel N., Guiriec B. (1997). Méthémoglobinémie Par Inhalation de poppers. Réanimation Urgences.

[130] Saito T., Takeichi S., Osawa M., Yukawa N., Huang X.-L. (2000). A Case of Fatal Methemoglobinemia of Unknown Origin but Presumably Due to Ingestion of Nitrate. Int. J. Leg. Med..

[131] Chou T.D., Gibran N.S., Urdahl K., Lin E.Y., Heimbach D.M., Engrav L.H. (1999). Methemoglobinemia Secondary to Topical Silver Nitrate Therapy—A Case Report. Burns.

[132] Retornaz F., Retornaz K., Seux V., Cortes E., Auffray J., Soubeyrand J. (2001). MéthémoglobinémieRécidivante: Rechercher La PriseItérative de Poppers. Rev. Med. Interne.

[133] Chui J.S., Poon W.T., Chan K.C., Chan A.Y., Buckley T.A. (2005). Nitrite-Induced Methaemoglobinaemia—Aetiology, Diagnosis and Treatment. Anaesthesia.

[134] Bénéteau-Burnat B., Pernet P., Vaubourdolle M., Pelloux P., Casenove L. (2005). Hypermethemoglobinemia in a Substance Abuser. Am. J. Emerg. Med..

[135] Zerbo S., Spanò M., Albano G.D., Buscemi R., Malta G., Argo A. (2023). A Fatal Suicidal Sodium Nitrite Ingestion Determined Six Days After Death. J. Forensic Leg. Med..

[136] Maric P., Ali S.S., Heron L.G., Rosenfeld D., Greenwood M. (2008). Methaemoglobinaemia Following Ingestion of a Commonly Available Food Additive. Med. J. Aust..

[137] Granados A., Luisa Iglesias M., Carod C., Artigas B. (2006). Intoxicación Aguda Por Ingesta de Carne de Pollo. Med. Clin..

[138] Moos M., Schröder R., Lang M., Frauchiger B. (2009). Schwere Methämoglobinämie—Diagnostik, Therapie Und Pathophysiologie Am Beispiel Eines Falles. Anasthesiol. Intensiv. Notfallmed Schmerzther..

[139] Kergueno J., Robquin P., Hubert J.C., Bertho N., Fievet-Brochot M.L., Ecollan P. (2009). Méthémoglobinémie Par Intoxication Au «Poppers»: Intérêt de La Mesure Non Invasive de La Méthémoglobine En Préhospitalier: À Propos d’un Cas. J. Eur. Urgences.

[140] Castagno E., Versace A., Grasso G., Bianciotto M., Bosetti F., Urbino A. (2012). Methaemoglobinaemia Caused by the Ingestion of Poisoned Meat in a Romanian Community in Italy. Acta Paediatr..

[141] McCabe A., McCann B., Kelly P. (2012). Pop Goes the O_2_: A Case of Popper-Induced Methaemoglobinamia. BMJ Case Rep..

[142] Sheena Y., Baston E.L., Downs A., Chester D.L. (2012). A Sticky Situation: Methaemaglobinaemia in a Hand Trauma Patient. BMJ Case Rep..

[143] Wang R., Teng C., Zhang N., Zhang J., Conway G. (2013). A Family Cluster of Nitrite Poisoning, Suzhou City, Jiangsu Province, China, 2013. West. Pac. Surveill. Response J..

[144] Bernasconi B., Konrad C., Fischer S. (2015). Kasuistik—SchwereIntoxikation Nach Oraler Einnahme von Alkylnitrit (“poppers“). Anasthesiol. Intensiv. Notfallmed Schmerzther..

[145] Kofler T., Lippay K., Goekcimen M., Fasel D., Nickel C. (2014). Use of Poppers (Amyl Nitrite): Unpleasant Side Effects in a Brothel. Eur. J. Case Rep. Intern. Med..

[146] Wellershoff G. (2014). Potenziell Letale Methämoglobinämie Nach Ingestion von Alkylnitriten (“poppers“). Notf. Rett. Med..

[147] Dalaker V.M., Vallersnes O.M., Fosshaug L.E., Andersson K.S., Hovda K.E. (2015). En Ung Kvinne Som Drakk Streptestreagens. Tidsskr. Nor. Laegeforen.

[148] Çağlar A., Er A., Karaarslan U., Ulusoy E., Akgül F., İnci G., Köroğlu T.F., Duman M., Yılmaz D. (2016). Severe Methemoglo-binemia Due to Nitrite Intoxication in a Child Who Was Misdiagnosed with Sepsis. J. Pediatr. Emerg. Intensive Care Med..

[149] Saccomani M.D., Cavarzere P., Silvagni D., Corso S.D., Perlini S., Biban P. (2016). A 5-Month-Old Infant with Diffuse Cyanosis and No Other Symptoms. Pediatr. Ann..

[150] Jiranantakan T., Olson K.R., Tsutaoka B., Smollin C.G. (2016). Methemoglobinemia from Frozen-Dried Mudfish Contaminated with Sodium Nitrite. Clin. Toxicol..

[151] Spiteri A. (2016). The Blue Patient. Emerg. Med. J..

[152] Martínez de Zabarte Fernández J.M., García Íñiguez J.P., Domínguez Cajal M. (2018). Metahemoglobinemia En Lactantes Mayores de Un Año. Med. Clin..

[153] Lefevre T., Nuzzo A., Mégarbane B. (2018). Poppers-Induced Life-Threatening Methemoglobinemia. Am. J. Respir. Crit. Care Med..

[154] Neth M.R., Love J.S., Horowitz B.Z., Shertz M.D., Sahni R., Daya M.R. (2020). Fatal Sodium Nitrite Poisoning: Key Considerations for Prehospital Providers. Prehosp. Emerg. Care.

[155] Tournoud C., Boltz P., Paradis C., Vodovar D., Puskarczyk E. (2021). Suicide Par Ingestion de Sels de Nitrites: C’est Possible!. Toxicol. Anal. Clin..

[156] Runkle A., Block J., Haydar S. (2020). Man with Cyanosis and Altered Mental Status. Ann. Emerg. Med..

[157] Bakos Á., Bátyi A. (2021). Illékony Nitritszármazékok (“Popperek”) Által Okozott Methaemoglobinaemia. Orv. Hetil..

[158] Taus F., Pigaiani N., Bortolotti F., Mazzoleni G., Brevi M., Tagliaro F., Gottardo R. (2021). Direct and Specific Analysis of Nitrite and Nitrate in Biological and Non-Biological Samples by Capillary Ion Analysis for the Rapid Identification of Fatal Intoxica-tions with Sodium Nitrite. Forensic Sci. Int..

[159] Hwang C., Yeon S.H., Jung J., Na J.Y. (2021). An Autopsy Case of Sodium Nitrite-Induced Methemoglobinemia with Various Post-Mortem Analyses. Forensic Sci. Med. Pathol..

[160] Tello D.M., Doodnauth A.V., Patel K.H., Gutierrez D., Dubey G.R. (2021). Poppers-Induced Methemoglobinemia: A Curious Case of the Blues. Cureus.

[161] Wettstein Z.S., Yarid N.A., Shah S. (2022). Fatal Methaemoglobinemia Due to Intentional Sodium Nitrite Ingestion. BMJ Case Rep..

[162] Ha H., Kim M.K., Moon S., Kang M. (2022). Fatal Nitrite Intoxication by Pickling Salt: Four Autopsy Cases. Korean J. Leg. Med..

[163] Chen Y., Liu Q., Wang J., Li H., Zhang Y., Sun L., Liu J. (2022). Delayed Post-Hypoxic Leukoencephalopathy Following Nitrite Poisoning: A Case Report and Review of the Literature. Front. Neurol..

[164] Sonck E., Bourmanne E., Bruteyn J., Dolip W. (2022). Methemoglobinemia Due to Use of Poppers: A Case Report. J. Med. Case Rep..

[165] Zhang M., Truver M.T., Hoyer J.L., Chronister C.W., Goldberger B.A. (2023). Presumptive Identification of Nitrite by Griess Reagent Test Strips—Case Reports of Fatal Poisoning with Sodium Nitrite. J. Anal. Toxicol..

[166] Sajko N., Finn K., Hill J., Khaira G.K., Duff J.P., Jiwani F., Allain D., Oliva M.A. (2022). Near-Fatal Pediatric Methemoglobinemia Secondary to Intentional Sodium Nitrite Ingestion. Am. J. Emerg. Med..

[167] Fan A.M., Steinberg V.E. (1996). Health Implications of Nitrate and Nitrite in Drinking Water: An Update on Methemoglobinemia Occurrence and Reproductive and Developmental Toxicity. Regul. Toxicol. Pharmacol..

[168] Geerdink R.B., Niessen W.M., Brinkman U.A. (2002). Trace-level determination of pesticides in water by means of liquid and gas chromatography. J. Chromatogr. A.

[169] Szpot P., Buszewicz G., Jurek T., Teresiński G. (2018). Fragmentation patterns involving ammonium adduct fragment ions: A comparison of the determination of metaldehyde in human blood by HPLC-QqQ-MS/MS and UHPLC-Q-TOF-MS. J. Chromatogr. B Anal. Technol. Biomed. Life Sci..

[170] Hernandez F., Beltran J., Lopez F.J., Gaspar J.V. (2000). Use of solid-phase microextraction for the quantitative determination of herbicides in soil and water samples. Anal. Chem..

[171] von Ehrenstein O.S., Ling C., Cui X., Cockburn M., Park A.S., Yu F., Wu J., Ritz B. (2019). Prenatal and infant exposure to ambient pesticides and autism spectrum disorder in children: Population based case-control study. BMJ.

[172] de Campos E.G., da Costa B.R.B., Dos Santos F.S., Monedeiro F., Alves M.N.R., Santos Junior W.J.R., De Martinis B.S. (2022). Alternative matrices in forensic toxicology: A critical review. Forensic Toxicol..

[173] Iskierka M., Zawadzki M., Szpot P., Jurek T. (2021). Detection of Drugs in Postmortem Specimens of Blood, Vitreous Humor and Bone Marrow Aspirate. J. Anal. Toxicol..

[174] Szpot P., Nowak K., Wachełko O., Tusiewicz K., Chłopaś-Konowałek A., Zawadzki M. (2023). Methyl (S)-2-(1–7 (5-fluoropentyl)-1H-indole-3-carboxamido)-3,3-dimethylbutanoate (5F-MDMB-PICA) intoxication in a child with identifica-tion of two new metabolites (ultra-high-performance liquid chromatography–tandem mass spectrometry). Forensic Toxicol..

[175] Szpot P., Wachełko O., Zawadzki M. (2022). Forensic Toxicological Aspects of Misoprostol Use in Pharmacological Abortions. Molecules.

[176] Wachełko O., Szpot P., Tusiewicz K., Nowak K., Chłopaś-Konowałek A., Zawadzki M. (2023). An ultra-sensitive UHPLC-QqQ-MS/MS method for determination of 54 benzodiazepines (pharmaceutical drugs, NPS and metabolites) and z-drugs in biological samples. Talanta.

[177] Szpot P., Wachełko O., Zawadzki M. (2022). Diclofenac Concentrations in Post-Mortem Specimens-Distribution, Case Reports, and Validated Method (UHPLC-QqQ-MS/MS) for Its Determination. Toxics.

[178] Tsikas D. (2005). Methods of quantitative analysis of the nitric oxide metabolites nitrite and nitrate in human biological fluids. Free Radic. Res..

[179] Grau M., Hendgen-Cotta U.B., Brouzos P., Drexhage C., Rassaf T., Lauer T., Dejam A., Kelm M., Kleinbongard P. (2007). Recent methodological advances in the analysis of nitrite in the human circulation: Nitrite as a biochemical parameter of the l-arginine/NO pathway. J. Chromatogr. B Anal. Technol. Biomed. Life Sci..

[180] Wang Q.H., Yu L.J., Liu Y., Lin L., Lu R., Zhu J., He L., Lu Z.L. (2017). Methods for the detection and determination of nitrite and nitrate: A review. Talanta.

[181] Wu A., Duan T., Tang D., Zheng Z., Zhu J., Wang R., He B., Cheng H., Feng L., Zhu Q. (2014). Review the Application of Chromatography in the Analysis of Nitric Oxide-derived Nitrite and Nitrate Ions in Biological Fluids. Curr. Anal. Chem..

[182] Suzuki O., Watanabe K., Okamoto N., Nozawa H., Ishii A. (2005). Simultaneous analysis of nitrite and nitrate in whole blood by ion chromatography. J. Liq. Chromatogr. Relat. Technol..

[183] Timerbaev A.R. (2008). Inorganic analysis of biological fluids using capillary electrophoresis. J. Sep. Sci..

[184] Kubáň P., Dvořák M., Kubáň P. (2019). Capillary electrophoresis of small ions and molecules in less conventional human body fluid samples: A review. Anal. Chim. Acta.

[185] He X., Mei Y., Wang Y., Sun W., Shen M. (2019). Determination of inorganic anions in the whole blood by ion chromatography. J. Pharm. Biomed. Anal..

[186] Akyüz M., Ata Ş. (2009). Determination of low level nitrite and nitrate in biological, food and environmental samples by gas chro-matography-mass spectrometry and liquid chromatography with fluorescence detection. Talanta.

[187] Jackson S.J., Siervo M., Persson E., McKenna L.M., Bluck L.J.C. (2008). A novel derivative for the assessment of urinary and salivary nitrate using gas chromatography/mass spectrometry. Rapid Commun. Mass. Spectrom..

[188] Tsikas D. (2000). Simultaneous derivatization and quantification of the nitric oxide metabolites nitrite and nitrate in biological fluids by gas chromatography/mass spectrometry. Anal. Chem..

[189] Jobgen W.S., Jobgen S.C., Li H., Meininger C.J., Wu G. (2007). Analysis of nitrite and nitrate in biological samples using high-performance liquid chromatography. J. Chromatogr. B Anal. Technol. Biomed. Life Sci..

[190] Li H., Meininger C.J., Wu G. (2000). Rapid determination of nitrite by reversed-phase high-performance liquid chromatography with fluorescence detection. J. Chromatogr. B Biomed. Sci. Appl..

[191] Tsikas D. (2007). Analysis of nitrite and nitrate in biological fluids by assays based on the Griess reaction: Appraisal of the Griess reaction in the l-arginine/nitric oxide area of research. J. Chromatogr. B Anal. Technol. Biomed. Life Sci..

[192] Brizzolari A., Dei Cas M., Cialoni D., Marroni A., Morano C., Samaja M., Paroni R., Rubino F.M. (2021). High-throughput griess assay of nitrite and nitrate in plasma and red blood cells for human physiology studies under extreme conditions. Molecules.

[193] Giustarini D., Rossi R., Milzani A., Dalle-Donne I. (2008). Nitrite and Nitrate Measurement by Griess Reagent in Human Plasma: Evaluation of Interferences and Standardization. Methods Enzym..

[194] Ricart-Jané D., Llobera M., López-Tejero M.D. (2002). Anticoagulants and other preanalytical factors interfere in plasma ni-trate/nitrite quantification by the Griess method. Nitric Oxide.

[195] Romitelli F., Santini S.A., Chierici E., Pitocco D., Tavazzi B., Amorini A.M., Lazzarino G., di Stasio E. (2007). Comparison of nitrite/nitrate concentration in human plasma and serum samples measured by the enzymatic batch Griess assay, ion-pairing HPLC and ion-trap GC-MS: The importance of a correct removal of proteins in the Griess assay. J. Chromatogr. B Anal. Technol. Biomed. Life Sci..

[196] Chophi R., Sharma S., Sharma S., Singh R. (2019). Forensic entomotoxicology: Current concepts, trends and challenges. J. Forensic Leg. Med..

[197] Groth O., Franz S., Fels H., Krueger J., Roider G., Dame T., Musshoff F., Graw M. (2022). Unexpected results found in larvae samples from two postmortem forensic cases. Forensic Toxicol..

[198] Janeczek A., Zawadzki M., Szpot P., Niedźwiedź A. (2018). Marijuana intoxication in a cat. Acta Vet. Scand..

[199] Żak-Bochenek A., Siwińska N., Slowikowska M., Borowicz H., Szpot P., Zawadzki M., Niedźwiedź A. (2018). The detection of capsaicin and dihydrocapsaicin in horse serum following long-term local administration. BMC Vet. Res..

[200] Gwaltney-Brant S.M. (2016). Veterinary Forensic Toxicology. Vet. Pathol..

[201] Chłopaś-Konowałek A., Tusiewicz K., Wachełko O., Szpot P., Zawadzki M. (2022). A Case of Amphetamine and Methampheta-mine Intoxication in Cat. Toxics.

[202] Brown H.M., McDaniel T.J., Fedick P.W., Mulligan C.C. (2020). The current role of mass spectrometry in forensics and future pro-spects. Anal. Methods.

[203] Helmke S.M., Duncan M.W. (2007). Measurement of the NO metabolites, nitrite and nitrate, in human biological fluids by GC-MS. J. Chromatogr. B Anal. Technol. Biomed. Life Sci..

[204] Tsikas D. (2017). Pentafluorobenzyl bromide—A versatile derivatization agent in chromatography and mass spectrometry: I. Analysis of inorganic anions and organophosphates. J. Chromatogr. B Anal. Technol. Biomed. Life Sci..

[205] Tesch J.W., Reeig W.R., Severs R.E. (1976). Microdetermination of nitrates and nitrites in saliva, blood, water, and suspended par-ticulates in air by gas chromatography. J. Chromatogr..

[206] Smythe G.A., Matanovic G., Yi D., Duncan M.W. (1999). Trifluoroacetic anhydride-catalyzed nitration of toluene as an approach to the specific analysis of nitrate by gas chromatography-mass spectrometry. Nitric Oxide.

[207] Zhan S.-y., Qing S., Li L., Fan X.-h (2013). A simple and accurate method to determine nitrite and nitrate in serum based on high-performance liquid chromatography with fluorescence detection. Biomed. Chromatogr..

[208] Zhao J., Wang J., Yang Y., Lu Y. (2015). The determination of nitrate and nitrite in human urine and blood by high-performance liquid chromatography and cloud-point extraction. J. Chromatogr. Sci..

[209] Croitoru M.D. (2012). Nitrite and nitrate can be accurately measured in samples of vegetal and animal origin using an HPLC-UV/VIS technique. J. Chromatogr. B Anal. Technol. Biomed. Life Sci..

[210] Fernandez-Cancio M., Marıa Fernandez-Vitos E., Centelles J.J., Imperial S. (2001). Sources of interference in the use of 2,3-diaminonaphthalene for the fluorimetric determination of nitric oxide synthase activity in biological samples. Clin. Chim. Acta.

[211] Nussler A.K., Glanemann M., Schirmeier A., Liu L., Nüssler N.C. (2006). Fluorometric measurement of nitrite/nitrate by 2,3-diaminonaphthalene. Nat. Protoc..

[212] Gutzki F.M., Tsikas D., Alheid U., Frolicht J.C. (1992). Determination of endothelium-derived nitrite/nitrate by gas chromatog-raphy/tandem mass spectrometry using (^15^N)NaNO_2_ as internal standard. Biol. Mass. Spectrom..

[213] Green L.C., Wagner D.A., Glogowski J., Skipper P.L., Wishnok J.S., Tannenbaum S.R. (1982). Analysis of nitrate, nitrite, and [^15^N]nitrate in biological fluids. Anal. Biochem..

[214] Tsikas D., Fuchs I., Gutzki F.M., Frolich J.C. (1998). Measurement of nitrite and nitrate in plasma, serum and urine of humans by high-performance liquid chromatography, the Griess assay, chemiluminescence and gas chromatography-mass spectrometry: Interferences by biogenic amines and N-nitro-L-arginine analogs. J. Chromatogr. B Biomed. Sci. Appl..

[215] Tsikas D. (2021). GC-MS analysis of biological nitrate and nitrite using pentafluorobenzyl bromide in aqueous acetone: A dual role of carbonate/bicarbonate as an enhancer and inhibitor of derivatization. Molecules.

[216] Kage S., Kudo K., Ikeda N. (2002). Simultaneous Determination of Nitrate and Nitrite in Human Plasma by Gas Chromatography-Mass Spectrometry. J. Anal. Toxicol..

[217] Yang X., Bondonno C.P., Indrawan A., Hodgson J.M., Croft K.D. (2013). An improved mass spectrometry-based measurement of NO metabolites in biological fluids. Free Radic. Biol. Med..

[218] Liu J.M., Liu C.C., Fang G.Z., Wang S. (2015). Advanced analytical methods and sample preparation for ion chromatography techniques. RSC Adv..

[219] Miyado T., Tanaka Y., Nagai H., Takeda S., Saito K., Fukushi K., Yoshida Y., Wakida S.I., Niki E. (2006). High-throughput nitric oxide assay in biological fluids using microchip capillary electrophoresis. J. Chromatogr. A.

[220] Wang X., Adams E., van Schepdael A. (2012). A fast and sensitive method for the determination of nitrite in human plasma by capillary electrophoresis with fluorescence detection. Talanta.

[221] Misko T.P., Schilling R.J., Salvemini D., Moore W.M., Currie M.G. (1993). A fluorometric assay for the measurement of nitrite in biological samples. Anal. Biochem..

[222] Duong D. (2022). Troubling Rise in Suicides Linked with Common Food Preservative. Can. Med. Assoc. J..

