# Supplementary material for: Nitrites: An Old Poison or a Current Hazard? Epidemiology of Intoxications Covering the Last 100 Years and Evaluation of Analytical Methods
Source: Toxics. 2023 Oct 1;11(10):832. doi: 10.3390/toxics11100832 (PMC10611400; doi:10.3390/toxics11100832)
Supplement: Supplementary file 1 [file toxics-11-00832-s001.zip › toxics-2623192-SP.pdf]

Supplementary Material

Review

Nitrites: an old poison or a current hazard? Epidemiology of intoxications covering the last 100 years and evaluation of analytical methods

Kaja Tusiewicz, Patryk Kuropka, Elżbieta Workiewicz, Olga Wachełko,
Paweł Szpot, Marcin Zawadzki

Abstract: In recent times, there has been a concerning and noteworthy rise in the global use of sodium nitrite for the suicidal purposes. These are facilitated either through the employment of specialized „suicide kits" or by acquiring sodium nitrite through alternative means. Additionally, another occurrence contributing to nitrite poisoning is the recreational utilization of nitrites in the form of volatile aliphatic esters of nitrous acid, commonly referred to as "poppers". Based on currently available papers and reports on the subject of nitrates, nitrites, and poppers intoxications, an epidemiological analysis was performed. A total of 130 papers, documenting a collective count of 492 intoxication cases, were identified. Additionally, in order to complete the epidemiological profile of nitrite poisoning, the authors briefly examined six cases of nitrite intoxication that were under investigation in our laboratory. Furthermore, a review of nitrite poisoning cases over the past 100 years shows that the old poison is still in use and poses a substantial risk to society.

Figure S1: Details regarding other approaches to nitrites and nitrated determination in biological material.

BATCH SPECTROPHOTOMETRIC ASSAYS

Giustarini2008

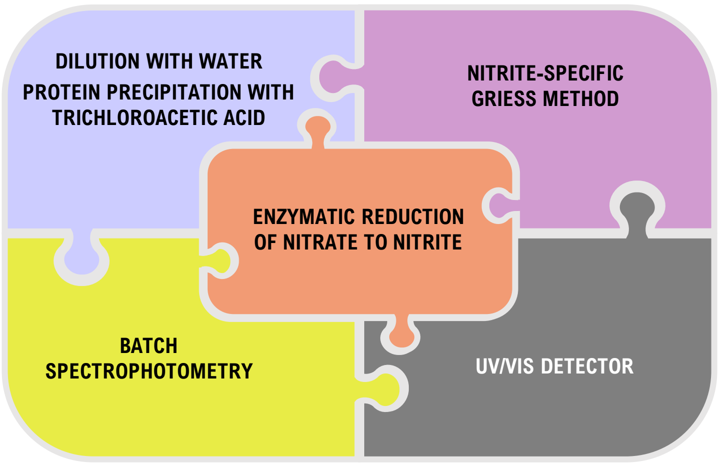

Brizzolari2021

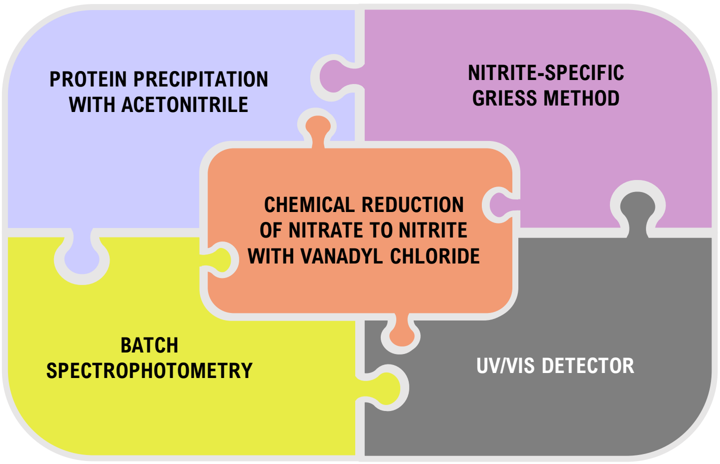

Simple and low-cost nitrite-specific colorimetric assays utilising a simple apparatus. Derivatisation is based on a popular reaction called the Griess method. This technique is prone to interferences and low sensitivity of analysis.

HPLC-UV ASSAYS

Chromatographic assays using ion pair chromatography or Griess method derivatisation. Low specific detection is based on the UV absorption capability of the analyte.

Wu2014

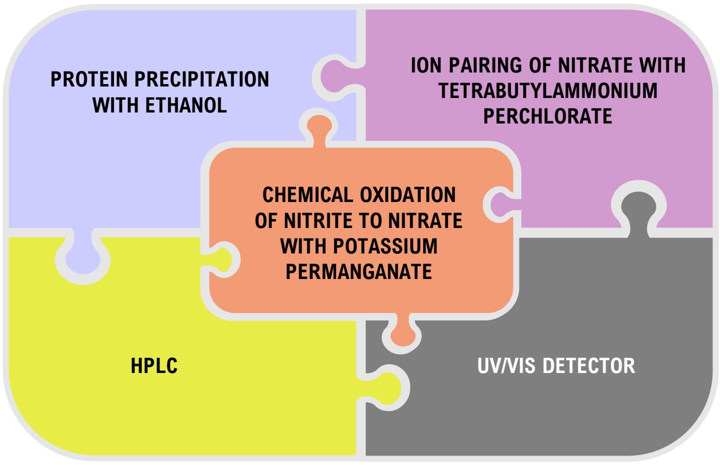

Croitoru2012

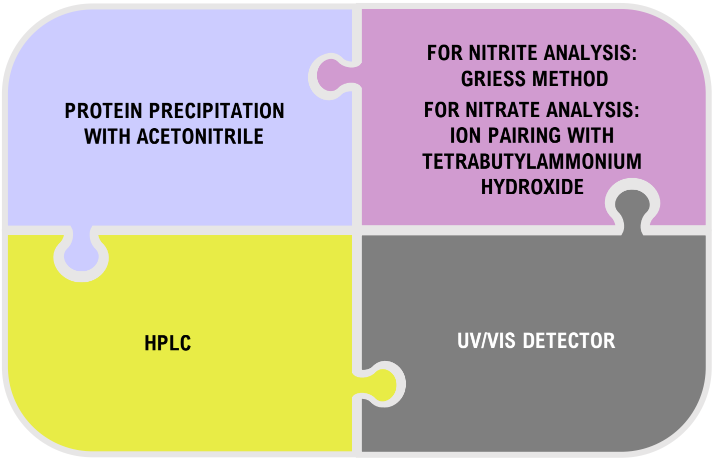

FLUORESCENCE ASSAYS

Misko1993

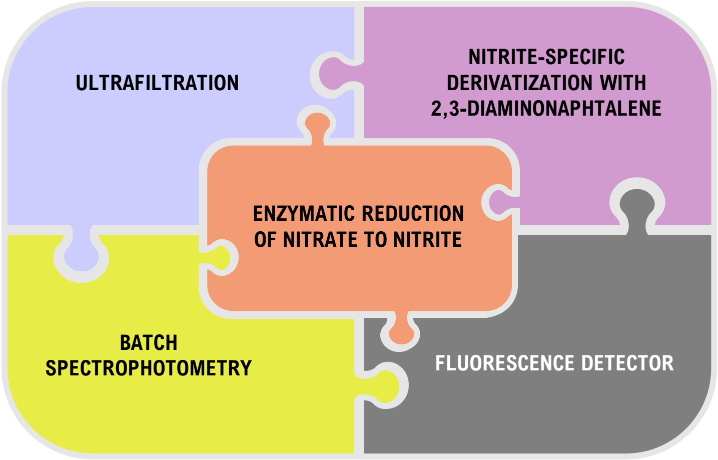

Akyuz2009

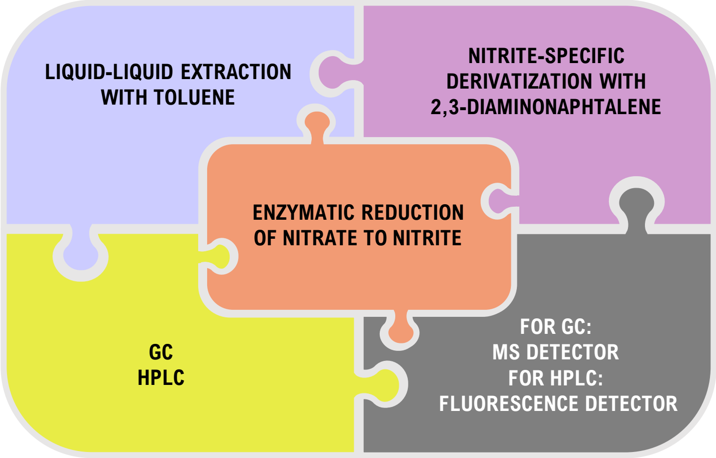

Li2000

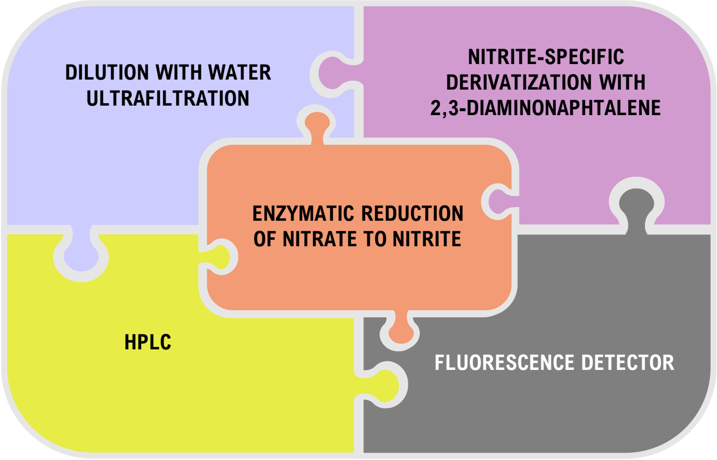

Batch or chromatographic assays using nitrite-specific derivatisation with the reagent 2,3-diaminonaphtalene. Detection based on the fluorescence properties of the resulting product.

NITRATION

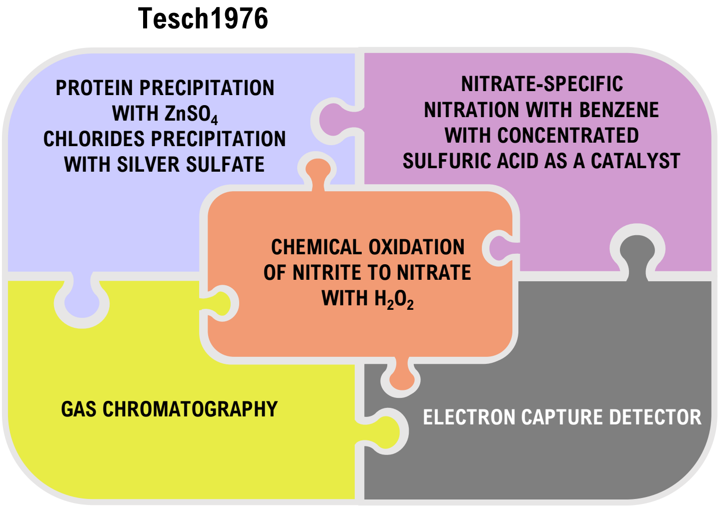

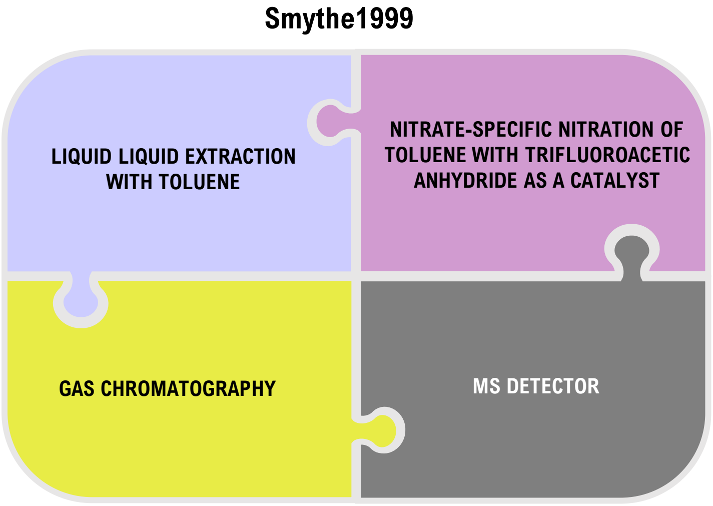

Gas chromatographic assays using nitrate-specific derivatisation with an aromatic compound in a strongly acidic medium. The resulting volatile and thermally stable products can be determined using various gas chromatography-compatible detectors.

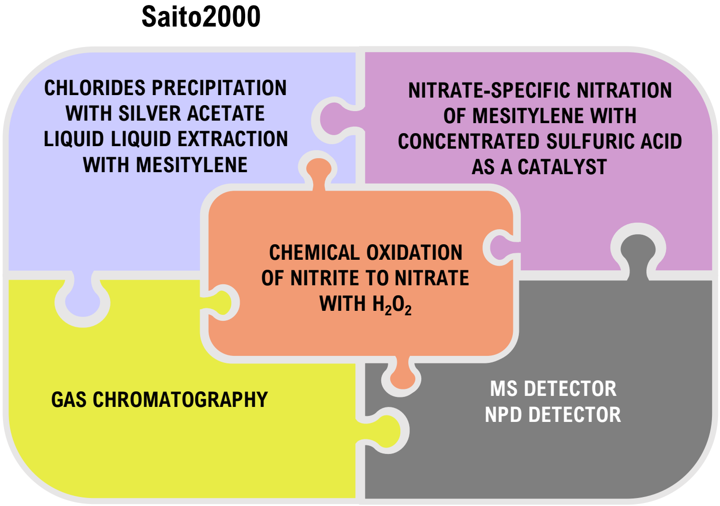

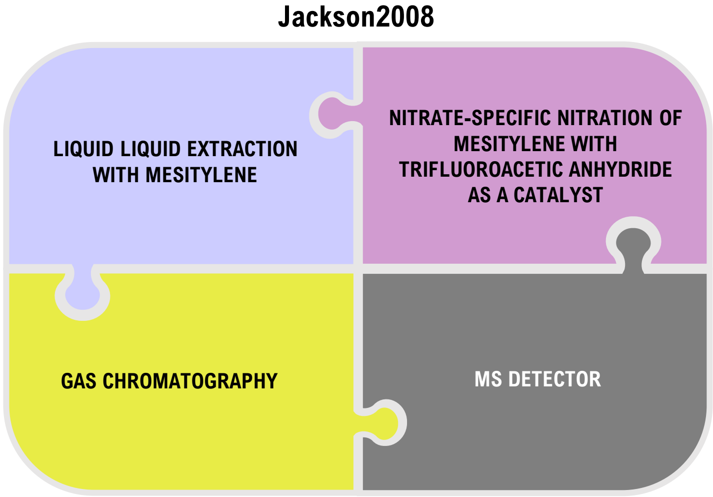

PFB-Br ALKILATION

Tsikas2000

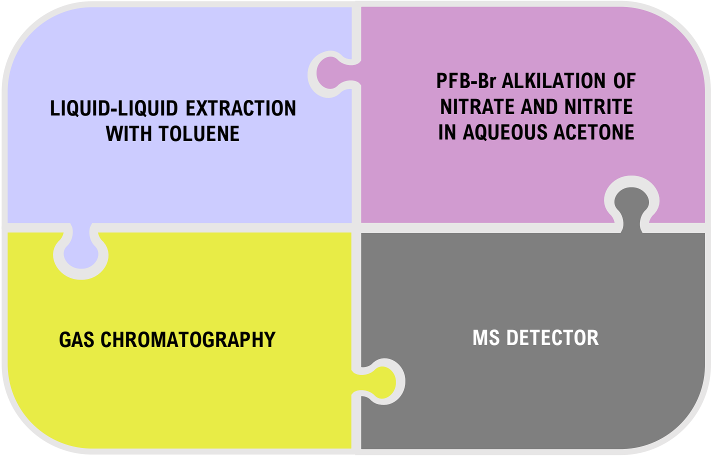

Kage2002

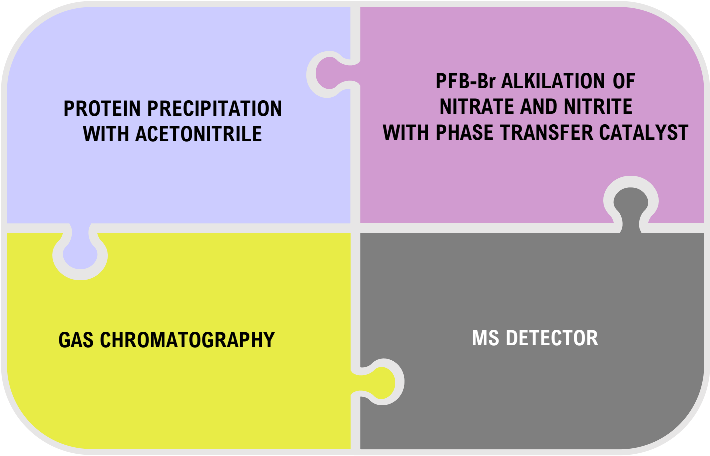

Gas chromatographic assays utilising derivatisation with PFB-Br reagent. Volatile and thermally stable products are formed, separate for nitrate and nitrite, which can be determined using a mass spectrometry detector.

CAPILLARY ELECTROPHORESIS

Taus2021

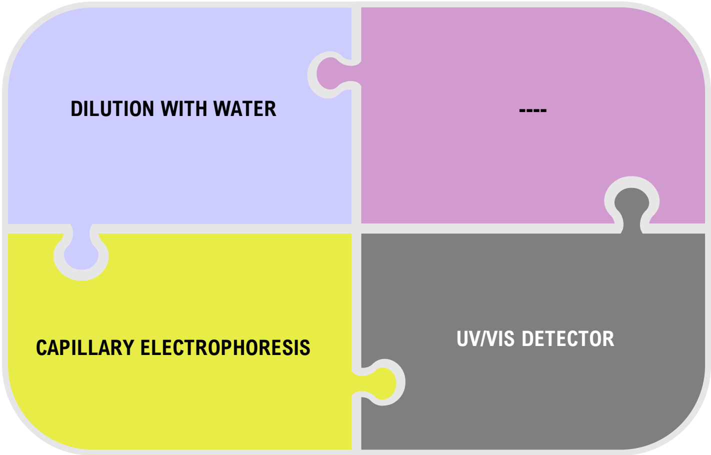

Determinations using electrophoretic mobility of anions. The use of a conductometric or UV/VIS detector allows direct determination of anions.

ION CHROMATOGRAPHY

Suzuki2005

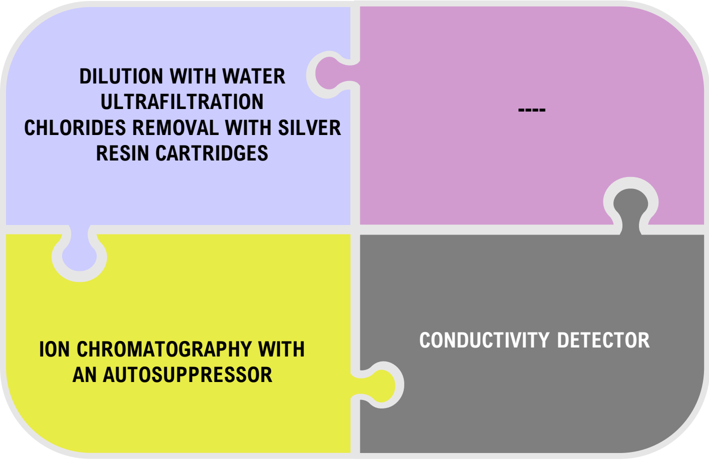

Kim2022

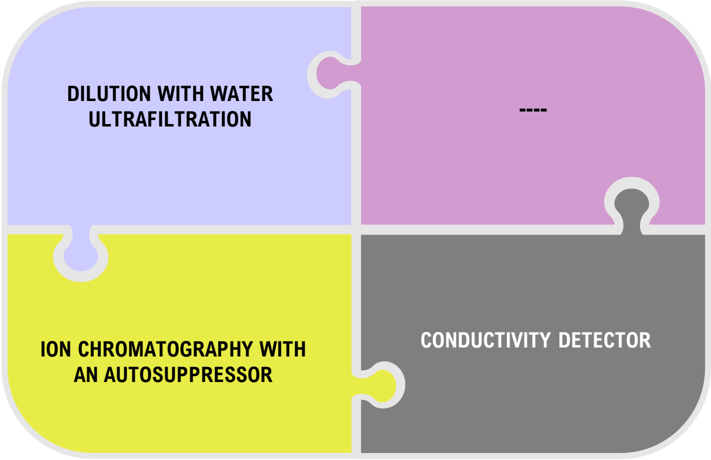

Yan2016

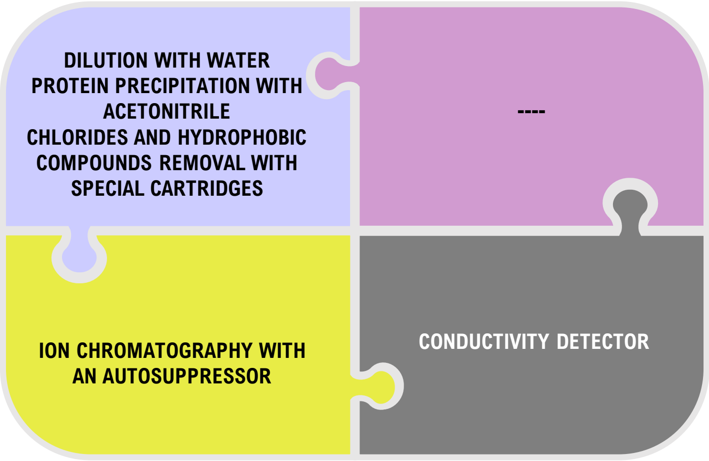

Ion chromatography assays. The use of a conductometric detector allows direct determination of anions.

Table S1: Measured concentrations of nitrite and/or nitrate in biological material in fatal poisoning cases.

Biological material	Nitrate [µg/ml]	Nitrite [µg/ml]	Method of nitrate/nitrite analysis	MetHb [%]	Reference
Serum<br>Gastric content	-<br>-	38<br>1 150	Spectrophotometric colorimetric method (Griess assay)	35	[60]
Peripheral blood<br>Vitreous humor<br>Gastric content	-<br>-<br>-	0.22<br>35<br>14 200	Spectrophotometric colorimetric method (Griess assay)	80	[40]
Blood<br>Gastric content	-<br>-	0.0246<br>93.91 mg/kg	NM	9.87	[71]
Blood<br>Gastric content	-<br>-	0.03<br>16 000	Spectrophotometric colorimetric method (Diazotization-coupling reaction)	-	[46]
Cardiac blood<br>Gastric content	-<br>-	35<br>8 800	Spectrophotometric colorimetric method (Griess assay)	-	[30]
Peripheral blood<br>Heart blood	39<br>45	1.7<br>1.6	NM	9	[162]
Peripheral blood<br>Heart blood	144.8<br>114.7	-<br>-	NM	8	
Peripheral blood<br>Heart blood	129<br>114	-<br>-	NM	10	
Peripheral blood<br>Heart blood	76.6<br>30.3	-<br>-	NM	9	
Blood<br>Gastric content<br>Lung<br>Liver	-<br>-<br>-<br>-	1.2<br>1.04 µg/g<br>0.931 µg/g<br>2.416 µg/g	IC	70.2	[103]
Blood<br>Gastric content<br>Lung<br>Liver	-<br>-<br>-<br>-	1.08<br>1.002 µg/g<br>0.907 µg/g<br>2.415 µg/g	IC	78.3	
Blood<br>Gastric content<br>Lung<br>Liver	-<br>-<br>-<br>-	0.83<br>0.962 µg/g<br>0.286 µg/g<br>2.324 µg/g	IC	87.4	
Peripheral blood<br>Heart blood<br>Pericardial fluid<br>Cerebrospinal fluid<br>Gastric content	220<br>218.5<br>91.7<br>50.5<br>137.8	-<br>-<br>181<br>-<br>11.2	IC	26	[159]

Heart blood	292	-	IC	-	[34]
Peripheral blood<br>Heart blood	236.9<br>131.5	8.1<br>3.5	IC	-	
Peripheral blood<br>Heart blood	363<br>201.6	1.2<br>1.3	IC	-	
Peripheral blood	319	298	IC	-	
Peripheral blood<br>Heart blood	476.4<br>524.8	9.4<br>8.4	IC	-	
Peripheral blood<br>Heart blood	340<br>277	7<br>170	IC	-	
Peripheral blood<br>Heart blood	220<br>218.5	-<br>-	IC	-	
Peripheral blood<br>Heart blood	237<br>212	-<br>-	IC	-	
Peripheral blood<br>Heart blood	282<br>369	-<br>-	IC	-	
Peripheral blood<br>Heart blood	213.8<br>172.7	-<br>-	IC	-	
Heart blood<br>Femoral blood	71.69<br>83.48	<0.05<br>0.09	Spectrophotometric colorimetric method (Griess assay)	83.4	[105]
Heart blood<br>Femoral blood	24<br>68	-<br>-	NM	30	[35]
Heart blood<br>Gastric content	292<br>200	34<br>11 000	NM	34	
Heart blood<br>Femoral blood<br>Gastric content	81.3<br>231.42<br>380	-<br>-<br>1 010	NM	43	
Heart blood<br>Femoral blood<br>Gastric content	171<br>230<br>180	19<br>10<br>4 180	NM	49	
Heart blood<br>Femoral blood<br>Gastric content	100.8<br>99.6<br>5 000	1.6<br>1.1<br>6 800	NM	-	
Heart blood<br>Gastric content	170.8<br>85	-<br>11 126	NM	-	
Femoral blood	288	51.0	NM	-	
Heart blood	92.2	8.4	NM	-	

Femoral blood	68.8	5.2			
Heart blood<br>Femoral blood<br>Gastric content	180<br>175.3<br>337.3	4 180<br>1.5<br>1.1	NM	-	
Heart blood<br>Femoral blood<br>Gastric content	1 470<br>377.9<br>462.6	2 220<br>2.5<br>2.1	NM	-	
Heart blood<br>Femoral blood<br>Gastric content	194<br>177<br>647	318<br>-<br>59 230	NM	-	
Heart blood<br>Femoral blood<br>Gastric content	162<br>475<br>167	<1<br><1<br>2 090	NM	-	
Heart blood<br>Femoral blood<br>Gastric content	1 130<br>90<br>10 590	175<br><1.0<br>67 400	NM	82.9 (AM)	
Heart blood<br>Femoral blood<br>Gastric content	8<br>271.3<br>2 486	43.7<br>0.1<br>9 000	NM	83 (AM)<br>41.6 (PM)	
Blood	1.5	0.76	GC-MS	78	[130]
Blood	4.4 mmol/L	-	CE	-	[158]
Blood	6.5 mmol/L	-	CE	-	
Blood<br>Urine<br>Gastric content<br>Liver<br>Costal cartilage<br>Vitreous humor	-<br>-<br>-<br>-<br>-<br>-	0.2<br>24.6<br>2 200<br>0.3 µg/g<br>3.4 µg/g<br>57.7	Spectrophotometric colorimetric method (Griess assay)	-	[41]

PM – post mortem, AM – ante mortem, IC – ion chromatography, CE – capillary electrophoresis, GC-MS – gas chromatography coupled with mass spectrometry, NM – not mentioned in the article
